# Activation of the Lateral Habenula‐Ventral Tegmental Area Neural Circuit Contributes to Postoperative Cognitive Dysfunction in Mice

**DOI:** 10.1002/advs.202202228

**Published:** 2022-05-26

**Authors:** Juan Xin, Weiran Shan, Jun Li, Hai Yu, Zhiyi Zuo

**Affiliations:** ^1^ Department of Anesthesiology University of Virginia Charlottesville VA 22908 USA; ^2^ Department of Anesthesiology West China Hospital Sichuan University Chengdu Sichuan 610041 China

**Keywords:** chemogenetic inhibition, endoplasmic reticulum stress, lateral habenula, postoperative cognitive dysfunction, ventral tegmental area

## Abstract

Postoperative cognitive dysfunction (POCD) is common and is associated with poor outcome. Neural circuit involvement in POCD is unknown. Lateral habenula (LHb) that regulates coping and depression‐like behaviors after aversive stimuli is activated by surgery in the previous study. Here, surgery activated LHb and ventral tegmental area (VTA) are presented. VTA is known to receive projections from LHb and project to the prefrontal cortex and hippocampus. Direct chemogenetic inhibition of LHb or damaging LHb attenuates surgery‐induced learning and memory impairment, *N*‐methyl‐d‐aspartate (NMDA) receptor activation, endoplasmic reticulum stress, inflammatory responses and cell injury in the VTA, and activation of rostromedial tegmental nucleus, an intermediate station to connect LHb with VTA. LHb inhibition preserves dendritic spine density in the prefrontal cortex and hippocampus. Retrograde inhibition of LHb via its projections to VTA attenuated surgery‐induced learning and memory dysfunction is observed. Retrograde activation of LHb induced learning and memory dysfunction is observed. Inhibition of NMDA receptors, dopamine synthesis, and endoplasmic reticulum stress in the VTA reduced surgery‐induced learning and memory impairment, inflammatory responses, and cell injury are observed. These results suggest that surgery activates the LHb‐VTA neural circuit, which contributes to POCD and neuropathological changes in the brain. These novel findings represent initial evidence for neural circuit involvement in surgery effects.

## Graphical abstract

The data suggests that the activation of the lateral habenula‐ventral tegmental area neural circuit is necessary and sufficient for postoperative cognitive dysfunction, a significant clinical syndrome affecting a large number of patients. Events subsequent to this circuit activation include glutamate receptor activation in the dopaminergic neurons and the increase of endoplasmic reticulum stress in the ventral tegmental area.

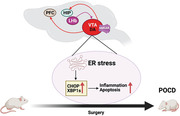

## 1 Introduction

Postoperative cognitive dysfunction (POCD) is a severe post‐surgery complication characterized by deterioration of memory and execution ability.^[^ ^1^, ^2^ ^]^ It can last several months, which seriously affects the quality of life of patients and places a heavy burden on the family and society.^[^ ^2^, ^3^ ^]^ Thus, there is an urgent need to identify effective interventions to reduce POCD. This need requires better understanding of the mechanisms of POCD.

POCD may involve dysfunction of multiple cognitive domains including working memory, long‐term memory, mental flexibility, selective attention, processing speed, and language processing. These functions involve cerebral cortex and various subcortical brain regions.^[^ ^4^ ^]^ However, basic science research mainly has focused on the changes in the hippocampus,^[^ ^5^ ^]^ a structure that is involved in long‐term memory.^[^ ^4^ ^]^ The role of other brain regions including neural circuits in POCD has not been reported.

Lateral habenula (LHb), a hub connecting the limbic forebrain and midbrain monoaminergic nuclei, modulates diverse physiological functions, including cognition, anxiety, sleep and pain processing.^[^ ^6^, ^7^, ^8^ ^]^ LHb receives strong inputs from the prefrontal cortex and contributes to value‐based decision‐making.^[^ ^9^ ^]^ In addition, LHb is adjacent to the hippocampus and interacts with the dorsal hippocampus to participate in the hippocampus‐dependent spatial information processing.^[^ ^10^ ^]^ Indeed, the spontaneous theta oscillation activity of the LHb is coupled with the hippocampal theta oscillation.^[^ ^11^ ^]^ Given the involvement in multiple cognitive functions and intimate interactions with the hippocampus, LHb may be involved in POCD. Consistent with this possibility, surgery activates LHb.^[^ ^12^ ^]^ Ventral tegmental area (VTA) is the major source of dopamine in the brain and has been linked to the pathogenesis of cognitive dysfunction.^[^ ^13^, ^14^, ^15^ ^]^ Dopamine neurons in the VTA project to the frontal cortex and hippocampus and regulate the development of dendritic spines in these brain regions.^[^ ^16^, ^17^, ^18^ ^]^ LHb projects to the VTA. Stimulating the projection fiber from LHb to VTA activates 48% of dopaminergic neurons in the VTA in a brain slice study^[^ ^19^ ^]^ but activating LHb can induce transient inhibition of dopaminergic neurons in the VTA via indirect connections between LHb and VTA.^[^ ^20^ ^]^ In addition, the LHb‐VTA circuit participates in coding responses to aversive stimuli.^[^ ^21^ ^]^ Thus, we hypothesize that the LHb‐VTA circuit contributes to the development of POCD. To test this hypothesis, we subjected adult mice to carotid artery exposure, a surgical procedure that does not affect motor function of limbs and the functions of major intra‐abdominal and intra‐thoracic organs. The role of the LHb‐VTA circuit in POCD was determined by inhibiting or injuring neurons in the LHb or VTA. Here, we showed that surgery activated the LHb‐VTA circuit. The activation of projections from LHb to VTA led to endoplasmic reticulum stress in the VTA. Chemogenetic inhibition of the LHb‐VTA circuit attenuated cognitive dysfunction after surgery. Our findings reveal the role of the LHb‐VTA circuit in POCD.

## 2 Results

### 2.1 Right Carotid Artery Exposure Surgery Activated LHb‐VTA Circuit and Impaired Cognitive Function

**Figure 1:**
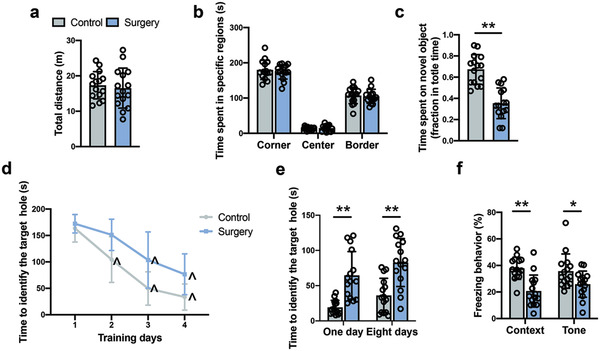
Surgery impaired learning and memory in mice. a,b) Performance in the open field test. c) Performance in the novel object recognition test. d) Performance in the training sessions of Barnes maze test. e) Performance in the memory phase of Barnes maze test. f) Performance in the context‐ and tone‐related fear conditioning test. Data are presented as mean ± S.D. with the presentation of data of each individual animal (*n* = 15). Results were analyzed by one‐way or two‐way repeated measures ANOVA (panel d) and *t*‐test (all other panels). *P < 0.05 for the comparison, **P < 0.01 for the comparison, ^*P* < 0.05 compared with the corresponding data on day 1.

There was no significant difference in total traveled distance and time spent in the corner, center and border areas between control and surgery mice in the open field test (**Figure** **1**a,b). These results suggest that mice in the control group and surgery group have similar locomotor activities. In the novel object recognition test, mice in the surgery group spent less time exploring a novel object compared with control group, indicating impaired cognitive function after surgery (Figure 1c). To explore their spatial learning and memory, mice were subjected to the Barnes maze test. As the number of training sessions was increased, the time for the two groups of mice to find the target hole in the maze was decreased compared to their corresponding time on the first day (Figure 1d). However, surgery was a significant factor to influence the performance of mice during the training sessions [F(1,27) = 19.20, *P* = 0.0002]. In addition, mice in the surgery group took a longer time than control mice to identify the target hole at 1 day and 8 days after the training sessions (Figure 1e). These results suggest that mice with surgery have poorer spatial learning and memory than control mice. To test the learning and memory of mice towards aversive stimuli, a fear conditioning experiment was conducted. The freezing behavior of mice with surgery was significantly shorter than control mice in the context‐ and tone‐related fear conditioning tests (Figure 1f). These results indicate that the right carotid artery exposure surgery impairs multiple domains of learning and memory in mice.

**Figure 2:**
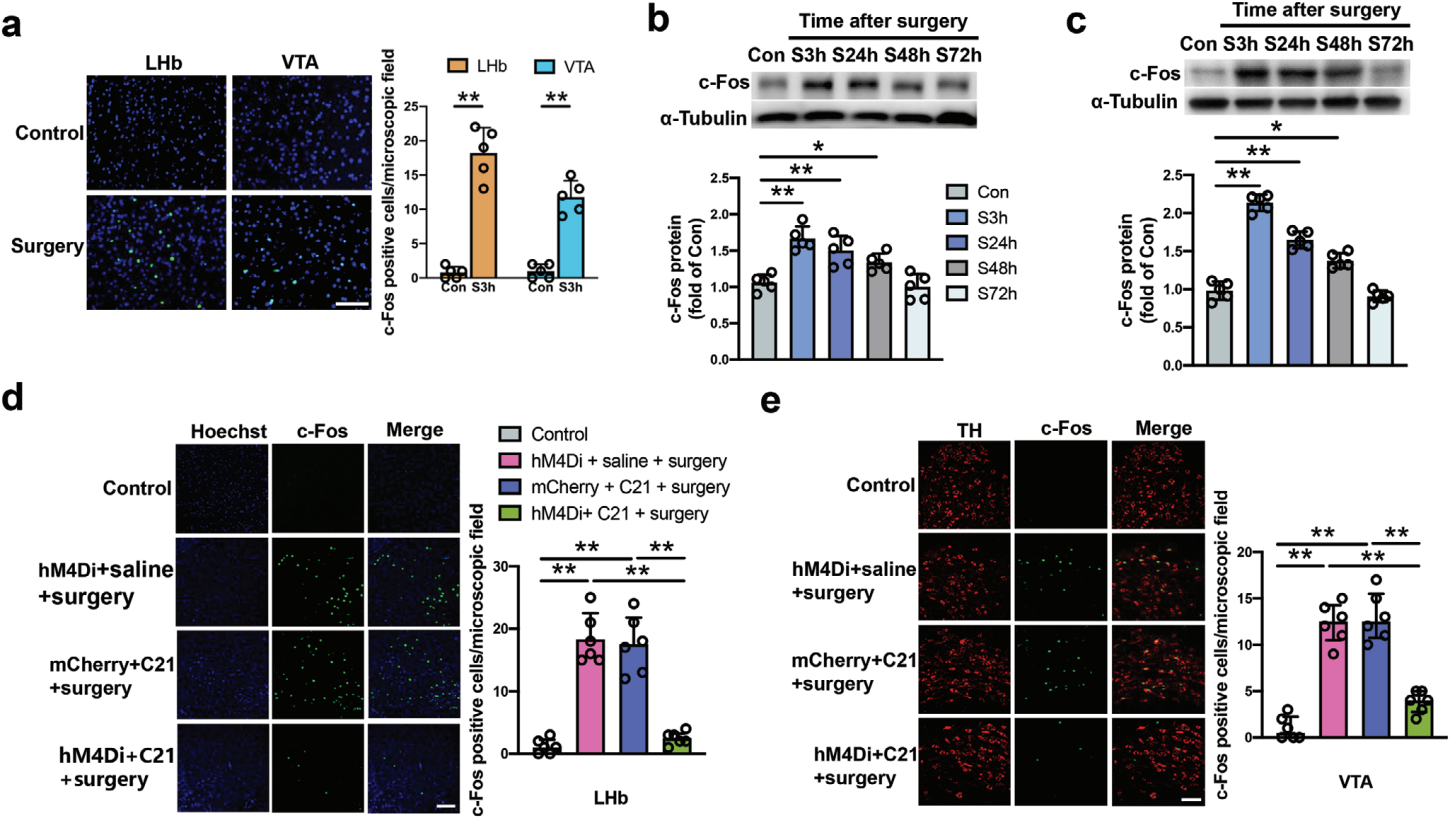
Activation of the LHb‐VTA circuit of mice with surgery and effectiveness of inhibition of this activation by a DREADDs approach. a) Left panel: Representative immunofluorescent images of c‐Fos staining (green) at 3 h (S3h) after surgery. Scale bar: 10 µm. Right panel: Quantitative data of the number of c‐Fos positive cells. b,c) Expression of c‐Fos in the LHb and VTA, respectively, at 3 h (S3h), 24 h (S24h), 48 h (S48h), or 72 h (S72h) after operation. d,e) Number of c‐Fos positive cells in the LHb and VTA, respectively, at 3 h after surgery. Left panel: Representative immunofluorescent images of c‐Fos staining. Scale bar: 100 µm. Right panel: quantitative data. Data are presented as mean ± S.D. with the presentation of data of each individual animal (*n* = 5 for panels (a–c) = 6 for panels (d,e). Results were analyzed by *t*‐test (panel a) and one‐way ANOVA followed with Tukey test (all other panels). * *P* < 0.05 for the comparison, ** *P* < 0.01 for the comparison.

To explore the activity of the LHb‐VTA circuit after surgery, c‐Fos staining was used to identify the neuronal activation 3 h after surgery. The number of c‐Fos positive cells was significantly increased in the LHb and VTA after surgery (**Figure** **2**a). The expression of c‐Fos protein in the LHb and VTA of mice with surgery remained high at 48 h after surgery and returned to the control level at 72 h after surgery (Figure 2b,c). To confirm the projection from LHb to VTA in our study, AAV2‐hSyn‐mCherry was injected into the LHb. Four weeks later, confocal microscopic imaging revealed the projections from the LHb to VTA. The projection fibers of LHb were co‐stained with vesicular glutamate transporter 2 (vGluT2), but not with vesicular GABA transporter (VGAT) (Figure S1a,b, Supporting Information). These results indicate that the LHb‐VTA circuit is activated after surgery and that the LHb projections to VTA are mainly from glutamatergic neurons. In addition, the retrograde virus carrying *gfp* code injected into the VTA also led to the expression of GFP in the LHb (Figure S2, Supporting Information), confirming that there are projections from the LHb to VTA. There was also GFP in the hippocampus and prefrontal cortex (PFC) after the injection of the retrograde virus into VTA (Figure S2, Supporting Information), suggesting that there are projections from the hippocampus and PFC to VTA.

### 2.2 Inhibition of the LHb‐VTA Circuit Attenuated Surgery‐Induced Learning and Memory Decline

**Figure 3:**
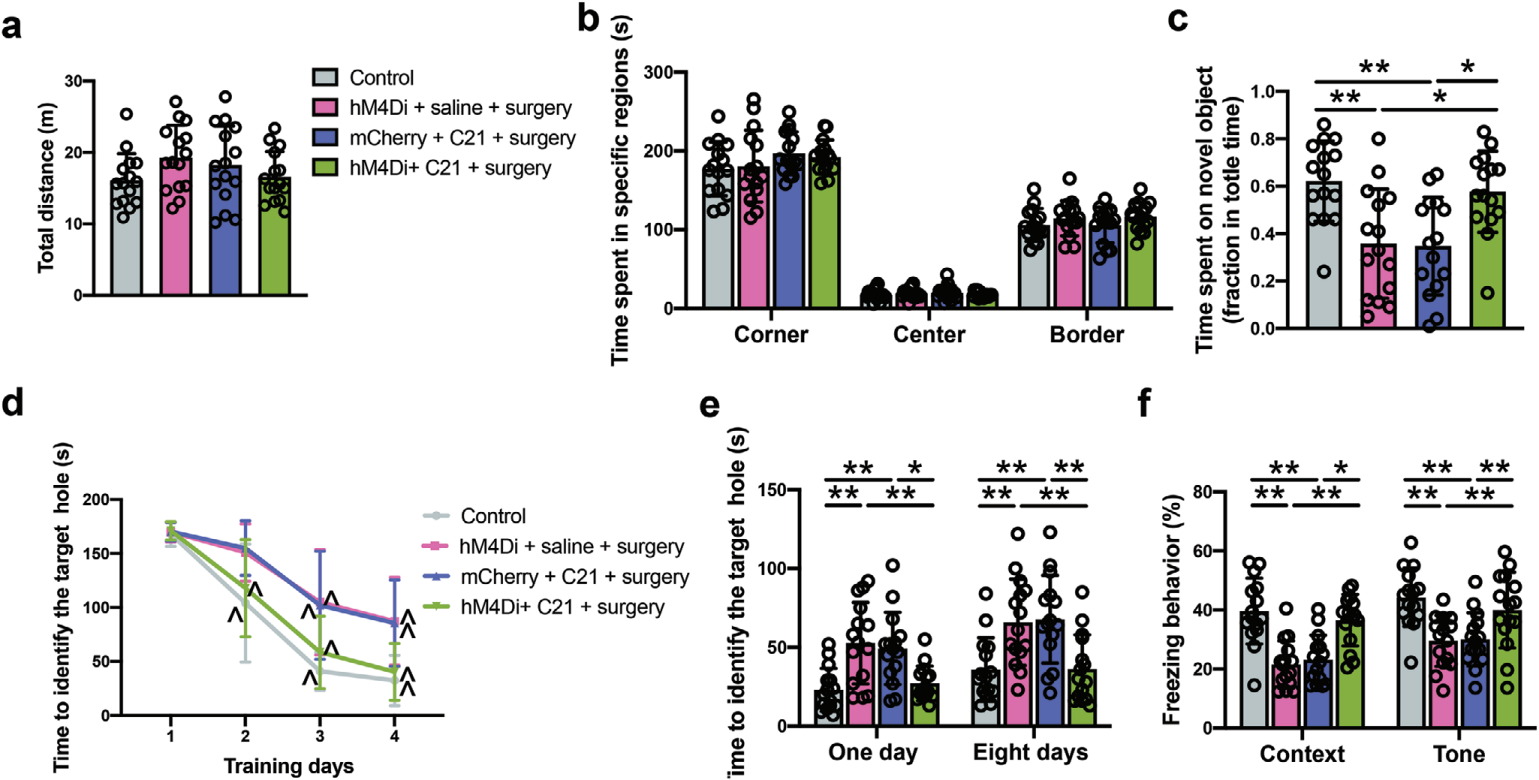
DREADDs‐based inhibition of the LHb‐VTA circuit attenuated learning and memory decline in mice with surgery. a,b) Performance in the open field test. c) Performance in the novel object recognition test. d) Performance in the training sessions of Barnes maze test. e) Performance in the memory phase of Barnes maze test. f) Performance in the context‐ and tone‐related fear conditioning test. Data are presented as mean ± S.D. with the presentation of data of each individual animal (*n* = 15). Results were analyzed by one‐way and two‐way repeated measures ANOVA (panel (d)) and one‐way ANOVA followed with Tukey test (all other panels). *P < 0.05 for the comparison, ** *P* < 0.01 for the comparison, ^*P* < 0.05 compared with the corresponding data on day 1.

To determine if activation of the LHb‐VTA circuit played a role in POCD, a chemogenetic approach was applied to inhibit neurons in the LHb. Since the use of chemogenetic and pharmacological approaches would involve injection of viruses or chemicals into the LHb and VTA, Evans blue was injected into these brain regions to examine the diffusion of solution. The staining of Evans blue was limited in these brain regions (Figure S3, Supporting Information), suggesting that the chemical does not diffuse to an area larger than intended after the injection. To apply the chemogenetic approach, mice received an injection of AAV2‐hsyn‐hM4Di‐mCherry or AAV2‐hsyn‐mCherry into the LHb. This injection led to transfections of more than 80% of the neurons in the LHb (Figure S1c, Supporting Information). Mice with surgery and receiving AAV2‐hsyn‐hM4Di‐mCherry and compound 21 had a decreased number of c‐Fos positive cells in the LHb and VTA compared with mice with surgery and receiving AAV2‐hsyn‐hM4Di‐mCherry and saline or receiving AAV2‐hsyn‐mCherry and compound 21. The number of c‐Fos positive cells in mice with surgery and injections of AAV2‐hsyn‐hM4Di‐mCherry and compound 21 was similar to that of control mice that did not receive surgery or injection of any solutions (Figure 2d,e). These results suggest that the designer receptors exclusively activated by designer drugs (DREADDs) approach effectively inhibits activation of the LHb‐VTA circuit in mice with surgery. It is known that LHb can project to the rostromedial tegmental nucleus (RMTg) that then projects to VTA.^[^ ^22^, ^23^ ^]^ As anticipated, the inhibition of the LHb neurons reduced the number of c‐Fos positive cells in the RMTg (Figure S4a,b, Supporting Information). Neurobehaviorally, inhibition of the LHb did not affect the performance of surgery mice in the open field test (**Figure** **3**a,b). However, this inhibition increased the time for mice to explore a new object after surgery (Figure 3c). Inhibition of the LHb was a significant factor to affect the performance of mice with surgery in the Barnes maze training sessions [F(1,28) = 26.56, *P* < 0.0001 for the comparison between surgery plus mCherry plus compound 21 group and surgery plus hM4Di plus compound 21 group] (Figure 3d). Inhibition of the LHb decreased the time for mice with surgery to identify the target hole at 1 day and 8 days after the training sessions (Figure 3e). The inhibition also increased the freezing behavior of surgery mice in the context‐related and tone‐related fear conditioning (Figure 3f). These results suggest that the activation of LHb mediates surgery‐induced learning and memory dysfunction.

**Figure 4:**
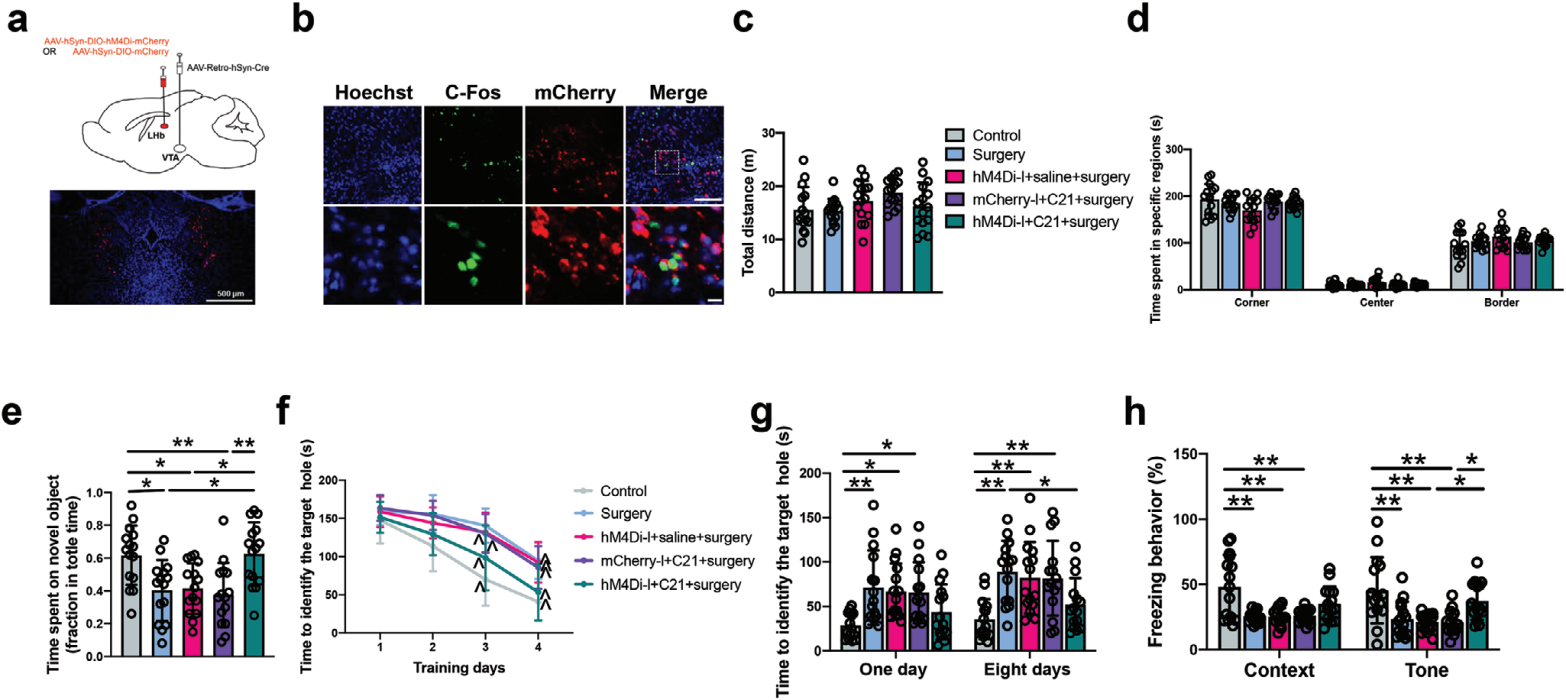
Retrograde inhibition of LHb neurons via the projections from LHb to VTA attenuated surgery‐induced learning and memory impairment. a) Top: schematic presentation of viral injections. Bottom: a) representative image of hM4Di‐mCherry expression in the LHb. Scale bar: 500 µm. b) Representative images of hM4Di‐transduced neurons and c‐Fos expression after surgery with intraperitoneal injection of saline (top panels, scale bar: 100 µm) and magnified images of a selected area shown in the top right image (scale bar: 10 µm) in the LHb. c,d) Performance in the open field test. e) Performance in the novel object recognition test. f) Performance in the training sessions of Barnes maze test. g) Performance in the memory phase of Barnes maze test. h) Performance in the context‐ and tone‐related fear conditioning test. Data are presented as mean ± S.D. with the presentation of data of each individual animal (*n* = 15). Results were analyzed by one‐way and two‐way repeated measures ANOVA (panel (f)) and one‐way ANOVA followed with Tukey test (all other panels). *P < 0.05 for the comparison, **P < 0.01 for the comparison, ^*P* < 0.05 compared with the corresponding data on day 1.

To determine the role of LHb‐VTA connection in POCD, mice received AAV2/2 retro plus‐hSyn‐Cre‐WPRE‐pA injection into the VTA and AAV2/9‐hSyn‐DIO‐hM4Di‐mCherry‐WPRE‐pA or AAV2/9‐hSyn‐DIO‐mCherry‐WPRE‐pA injection into the LHb (**Figure** **4**a,b). The application of compound 21 in mice receiving the injection of AAV2/2 retro plus‐hSyn‐Cre‐WPRE‐pA into the VTA and AAV2/9‐hSyn‐DIO‐hM4Di‐mCherry‐WPRE‐pA into the LHb would induce retrograde inhibition of neurons in the LHb. As anticipated, mice with surgery and this retrograde inhibition had a reduced number of c‐Fos positive cells in the LHb than mice with surgery but without the activation of the retrograde inhibition mechanisms (Figure S4a,c, Supporting Information). Consequently, the number of c‐Fos positive cells in the VTA and RMTg was decreased in mice with surgery and retrograde inhibition of the LHb neurons (Figure S4d,e, Supporting Information). Neurobehaviorally, there was no difference in the performance in the open field test among the five groups with various treatments (Figure 4c,d). However, mice with surgery and retrograde inhibition of neurons in the LHb spent more time to explore a new object than mice with surgery and without the inhibition (Figure 4e). Retrograde inhibition of neurons in the LHb was a significant factor to affect the performance of mice with surgery in the Barnes maze training sessions [F(1,28) = 33.716, *P* < 0.0001 for the comparison between surgery plus mCherry plus compound 21 group and surgery plus retrograde inhibition group] (Figure 4f). Retrograde inhibition decreased the time for mice with surgery to identify the target hole at 8 days after the training sessions (Figure 4g). This inhibition also increased the freezing behavior of mice with surgery in the tone‐related fear conditioning (Figure 4h). These results clearly suggest the importance of LHb‐VTA connections in surgery‐induced learning and memory dysfunction.

**Figure 5:**
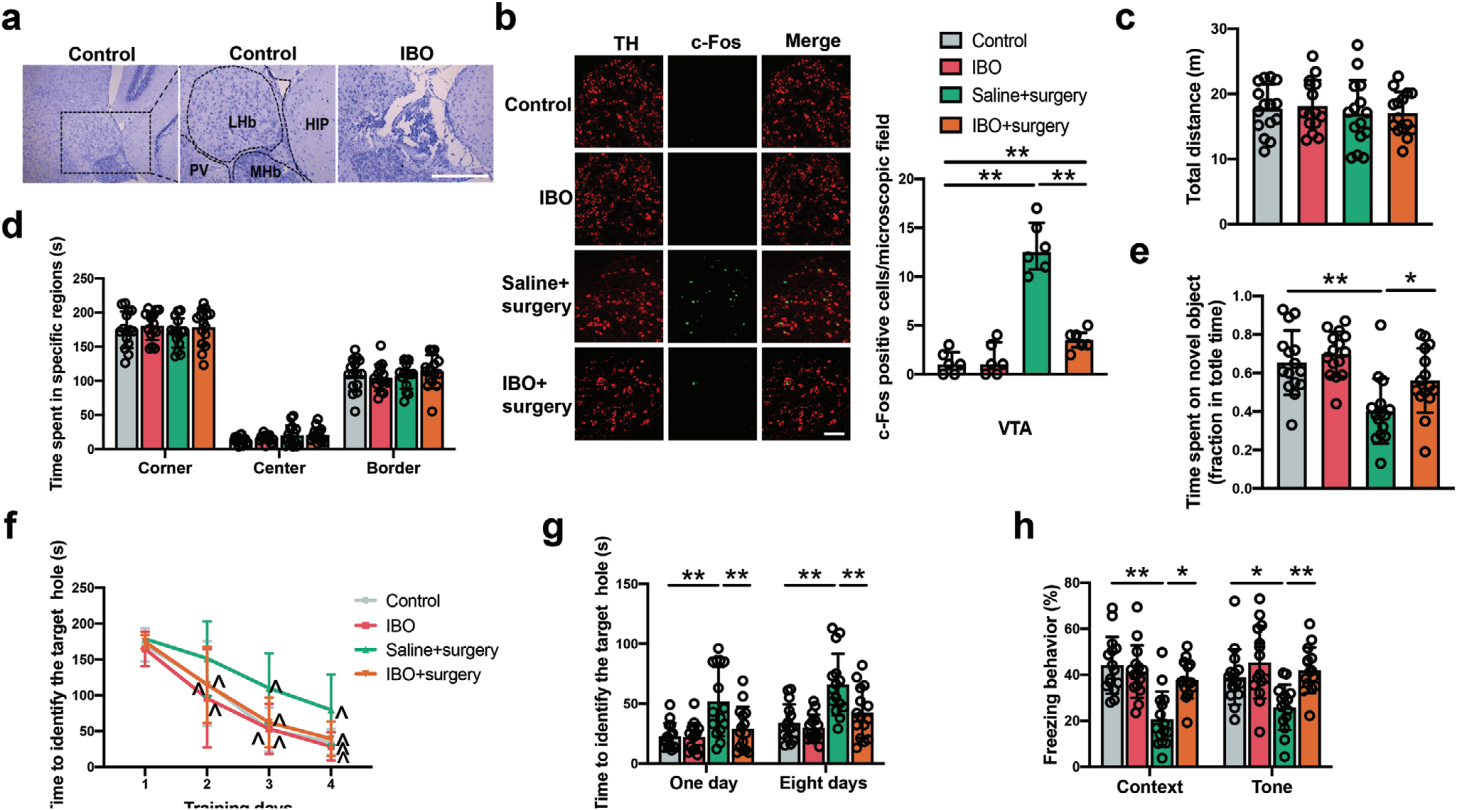
Chemical lesion of the LHb attenuated cognitive dysfunction in mice with surgery. a) Representative images of Nissl staining showing structural damage in the LHb. Scale bar: 100 µm. b) Left panel: Representative immunofluorescent images of c‐Fos in the VTA at 3 h after surgery. Scale bar: 100 µm. Right panel: Quantitative results of c‐Fos positive cells. c,d) Performance in the open field test. e) Performance in the novel object recognition test. f) Performance in the training sessions of Barnes maze test. g) Performance in the memory phase of Barnes maze test. h) Performance in the context‐ and tone‐related fear conditioning test. Data are presented as mean ± S.D. with the presentation of data of each individual animal (*n* = 6 for panel (b), = 15 for all other panels). Results were analyzed by one‐way and two‐way repeated measures ANOVA (panel (f)) and one‐way ANOVA followed with Tukey test (all other panels). *P < 0.05 for the comparison, **P < 0.01 for the comparison, ^*P* < 0.05 compared with the corresponding data on day 1. Hip: hippocampus; IBO: ibotenic acid; MHb: medial habenular nucleus; PV: paraventricular thalamic nucleus; TH: tyrosine hydrolase.

To supplement the approach of DREADDs, ibotenic acid, a commonly used neurotoxin to injure neurons but without damaging neighboring nerve fibers,^[^ ^24^ ^]^ was used to cause a LHb lesion. The injection of ibotenic acid into the LHb caused LHb injury (**Figure** **5**a). This injury inhibited the VTA and RMTg neuronal activation after surgery (Figure 5b; Figure S4f,g, Supporting Information) but did not affect the performance of mice in the open field test (Figure 5c,d). Although the LHb lesion did not affect the performance of mice without surgery in the novel object recognition test, this lesion increased the time of mice with surgery to explore a new object (Figure 5e). The LHb lesion improved the performance of mice after surgery in the training sessions of the Barnes maze test [F(1,28) = 21.49, *P* < 0.0001] (Figure 5f). Compared to mice with surgery, surgery mice receiving ibotenic acid needed less time to identify the target hole at 1 day and 8 days after the training sessions of Barnes maze test (Figure 5g). Surgery mice with the LHb lesion increased the freezing behavior in the context‐related and tone‐related fear conditioning (Figure 5h). These results, along with the results of DREADDs, strongly suggest the involvement of LHb‐VTA circuit activation in POCD.

### 2.3 Activation of LHb Neurons Induced Learning and Memory Dysfunction

**Figure 6:**
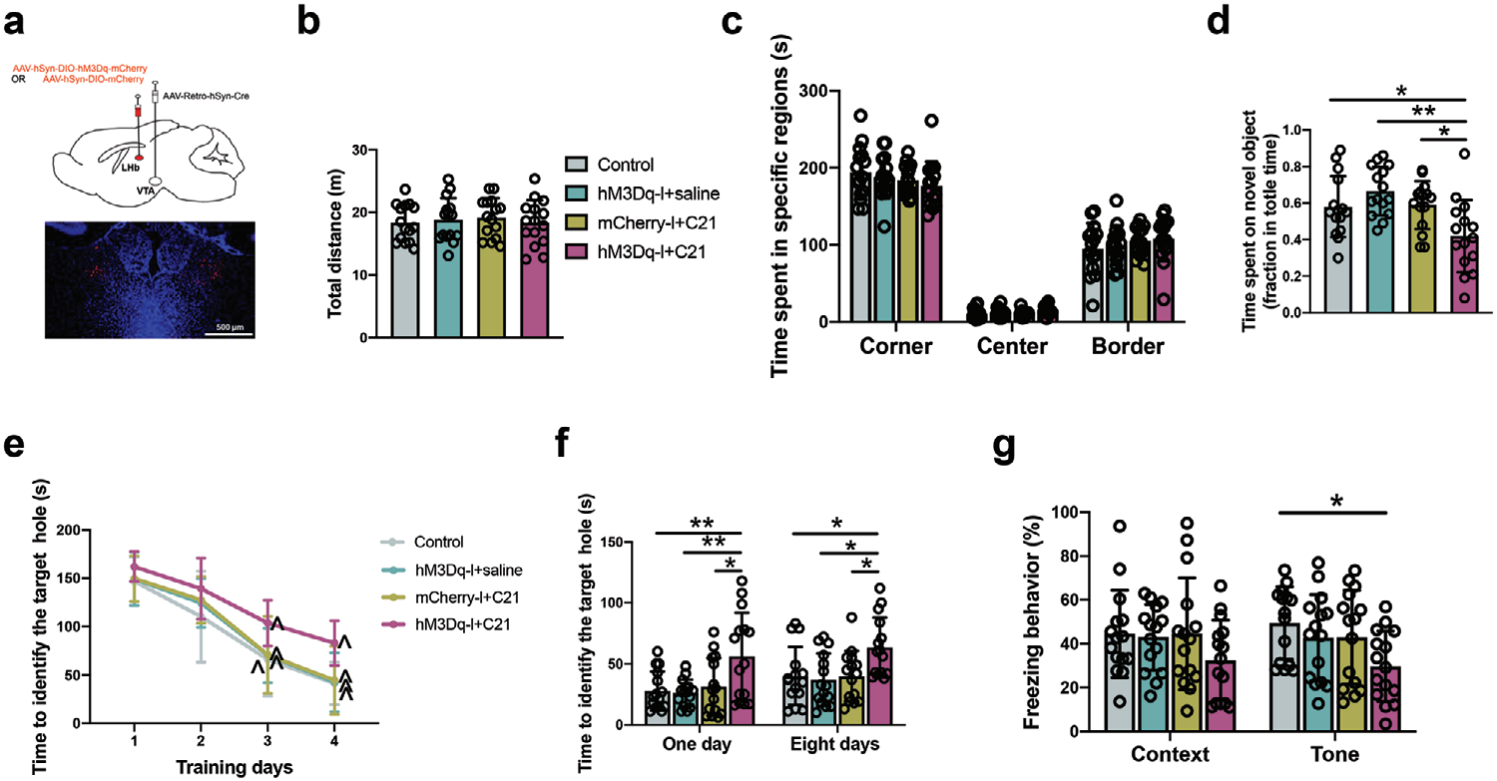
Chemogenetic activation of LHb neurons via the projections from LHb to VTA impaired learning and memory. a) Top: schematic presentation of the viral injections. Bottom: representative images of hM3Dq‐mCherry expression in the LHb. Scale bar: 500 µm). b,c) Performance in the open field test. d) Performance in the novel object recognition test. e) Performance in the training sessions of Barnes maze test. f) Performance in the memory phase of Barnes maze. g) Performance in the context‐ and tone‐related fear conditioning test. Data are presented as mean ± S.D. with the presentation of data of each individual animal (*n* = 15). Results were analyzed by one‐way and two‐way repeated measures ANOVA (panel (e)) and one‐way ANOVA followed with Tukey test (all other panels). *P < 0.05 for the comparison, **P < 0.01 for the comparison, ^*P* < 0.05 compared with the corresponding data on day 1.

To retrogradely activate the LHb neurons, mice received AAV2/2 retro plus‐hSyn‐Cre‐WPRE‐pA injection into the VTA and AAV2/9‐hSyn‐DIO‐hM3Dq‐mCherry‐WPRE‐pA injection into the LHb (**Figure** **6**a). Retrograde activation of the LHb neurons did not affect the performance of mice in the open field test (Figure 6b,c). However, mice with retrograde activation of neurons in the LHb spent less time to explore a new object than control mice or mice receiving virus injection but without compound 21 to induce the activation of LHb neurons (Figure 6d). Retrograde activation of neurons in the LHb was a significant factor to impair the performance of mice in the Barnes maze training sessions [F(1,28) = 29.467, *P* < 0.0001 for the comparison between mCherry plus compound 21 group and retrograde activation group] (Figure 6e). Retrograde activation increased the time for mice to identify the target hole at 1 day or 8 days after the training sessions (Figure 6f). This activation also decreased the freezing behavior of mice with surgery in the tone‐related fear conditioning (Figure 6g). These results suggest that the activation of LHb neurons via the LHb‐VTA connections is sufficient to induce learning and memory dysfunction.

### 2.4 Surgery Induced Endoplasmic Reticulum Stress in VTA via Activation of NMDA Receptors

**Figure 7:**
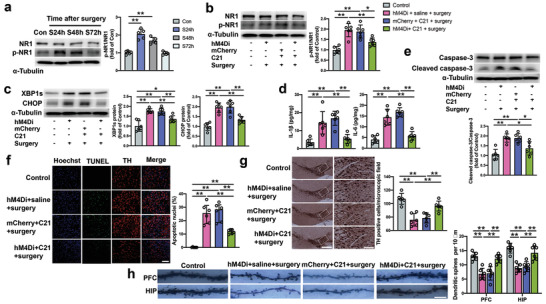
Inhibition of the LHb‐VTA circuit reduced NMDA receptor activation and endoplasmic reticulum stress in mice with surgery. a) Expression of p‐NR1 in the VTA at 24 h (S24h), 48 h (S48h), or 72 h (S72h) after surgery. Left panel: Representative images of Western blots. Right panel: Quantitative results. b,c) Expression of p‐NR1, XBP1s and CHOP in the VTA at 24 h after surgery. Left panel: Representative images of Western blots. Right panel: Quantitative results. d) Concentrations of IL‐1*β* and IL‐6 in the VTA at 24 h after surgery. e) Expression of caspase‐3 and cleaved caspase‐3 in the VTA at 24 h after surgery. Top panel: Representative images of Western blots. Bottom panel: Quantitative results. f) Number of TUNEL positive cells in the VTA at 24 h after surgery. Left panel: Representative images of TUNEL staining. Scale bar: 100 µm. Right panel: Quantitative data. g) Number of dopaminergic neurons in the VTA at 20 days after surgery. Left panel: Representative images of staining for tyrosine hydrolase (TH). Scale bar: 400 µm (left column) and 100 µm (right column). Right panel: Quantitative data of TH‐positive cells. h) Dendritic spine density in the prefrontal cortex (PFC) and hippocampus (HIP) at 20 days after surgery. Left panel: Representative images of Golgi staining. Scale bar: 5 µm. Right panel: Quantitative data. Data are presented as mean ± S.D. with the presentation of data of each individual animal (*n* = 5 for panel (a), = 6 for all other panels). Results were analyzed by one‐way ANOVA followed with Tukey test. * *P* < 0.05 for the comparison, ** *P* < 0.01 for the comparison.

To determine the molecular mechanism after the LHb‐VTA circuit activation for POCD, molecular changes in the VTA were examined. The phosphorylated NMDA receptor 1 (p‐NR1) level in the VTA was increased 24 and 48 h after surgery and returned to the control level at 72 h after the operation (**Figure** **7**a), which was consistent with the time‐course of activation of the LHb‐VTA circuit. DREADDs‐based inhibition of the LHb reduced the level of p‐NR1 in the VTA (Figure 7b). Considering that the LHb projections to VTA are mainly glutamatergic,^[^ ^25^ ^]^ p‐NR1 level is closely related to NMDA receptor activity^[^ ^26^ ^]^ and continuous excitation of NMDA receptors may cause excitotoxicity in VTA,^[^ ^27^ ^]^ endoplasmic reticulum stress was determined. The expression of endoplasmic reticulum stress‐related proteins, X‐box binding protein 1s (XBP1s) and CCAAT‐enhancer‐binding protein homologous protein (CHOP),^[^ ^28^, ^29^ ^]^ in the VTA was inhibited in surgery mice with LHb inhibition compared with that in surgery mice without LHb inhibition (Figure 7c). The inhibition of LHb reduced the levels of interleukin (IL)‐1*β*, IL‐6, and cleaved caspase‐3 in the VTA of mice with surgery (Figure 7d,e). There was a reduction in the number of terminal deoxynucleotidyl transferase dUTP nick end labeling (TUNEL) positive staining cells in the VTA of surgery mice with LHb inhibition (Figure 7f). The VTA is a brain area where dopaminergic neurons are concentrated.^[^ ^30^ ^]^ Consistent with the findings of reduced endoplasmic reticulum stress and neuropathological changes after LHb inhibition, this inhibition maintained the number of dopaminergic neurons in the VTA of surgery mice (Figure 7g). It is known that VTA releases dopamine to the PFC and hippocampus and that dopamine regulates the development of dendritic spines,^[^ ^16^, ^17^, ^18^ ^]^ structures that are involved in cognitive function. Our previous studies have shown that dendritic spine density is reduced in the hippocampus after a peripheral surgery.^[^ ^31^, ^32^ ^]^ Consistent with these previous findings, inhibition of the LHb‐VTA circuit maintained spine density in the PFC and hippocampus of surgery mice (Figure 7h). Taken together, these data suggest that activation of the LHb‐VTA circuit leads to endoplasmic reticulum stress, neuroinflammatory responses, cell injury and impairment of dendritic spine development after surgery.

### 2.5 Inhibition of NMDA Receptors in VTA Alleviated Surgery‐Induced Cognitive Decline through Reduction of Endoplasmic Reticulum Stress

**Figure 8:**
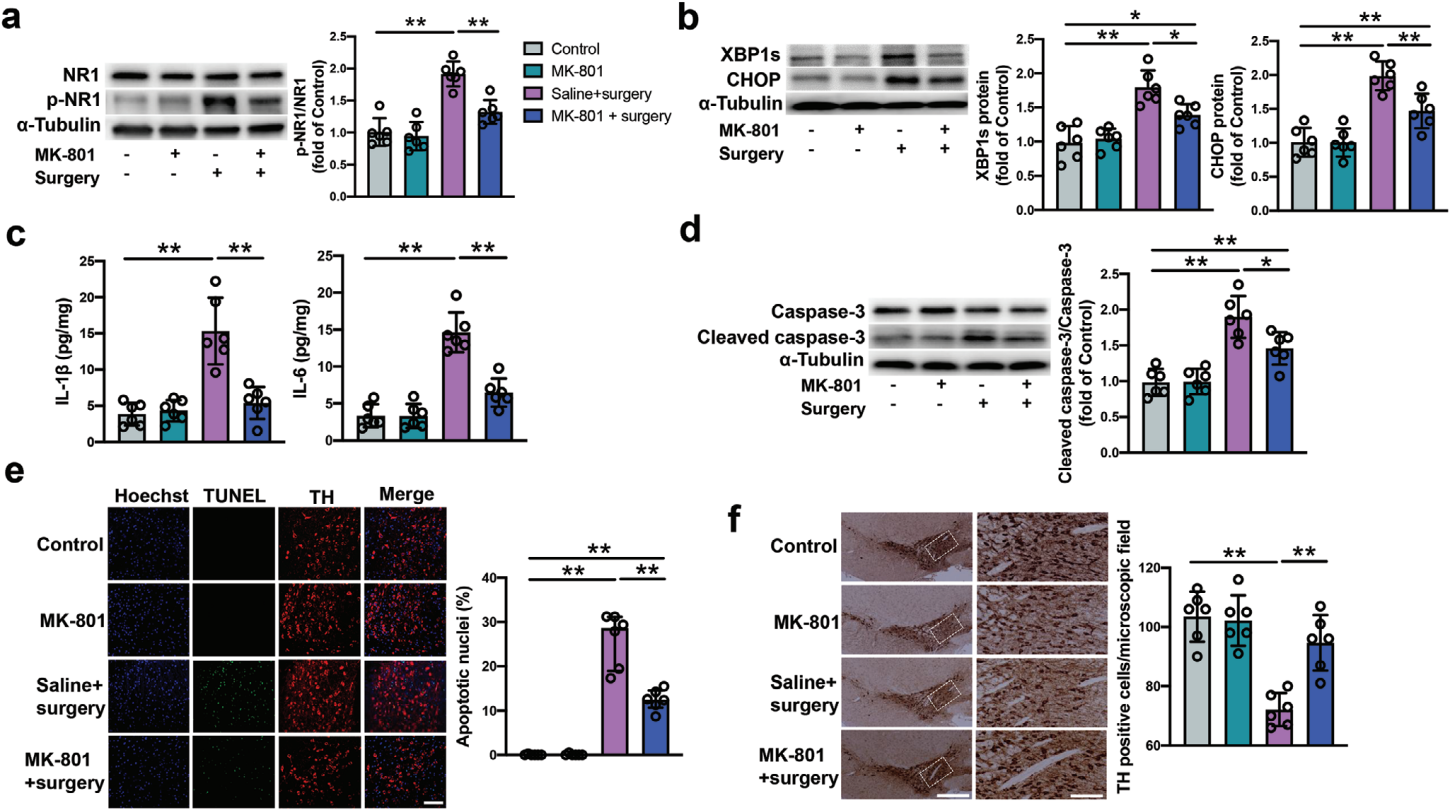
NMDA receptor inhibition by MK‐801 attenuated endoplasmic reticulum stress and neuropathological changes in mice with surgery. a,b) Expression of p‐NR1, XBP1s and CHOP in the VTA at 24 h after surgery. Left panel: Representative images of Western blots. Right panel: Quantitative results. c) Concentrations of IL‐1*β* and IL‐6 in the VTA at 24 h after surgery. d) Expression of caspase‐3 and cleaved caspase‐3 in the VTA at 24 h after surgery. Left panel: Representative images of Western blots. Right panel: Quantitative results. e) Number of TUNEL positive cells in the VTA at 24 h after surgery. Left panel: Representative images of TUNEL staining. Scale bar: 100 µm. Right panel: Quantitative data. f) Number of dopaminergic neurons in the VTA at 20 days after surgery. Left panel: Representative images of staining for tyrosine hydrolase (TH). Scale bar: 400 µm (left column) and 100 µm (right column). Right panel: Quantitative data of TH positive cells. Data are presented as mean ± S.D. with the presentation of data of each individual animal (*n* = 6). Results were analyzed by one‐way ANOVA followed with Tukey test. * *P* < 0.05 for the comparison, ** *P* < 0.01 for the comparison.

**Figure 9:**
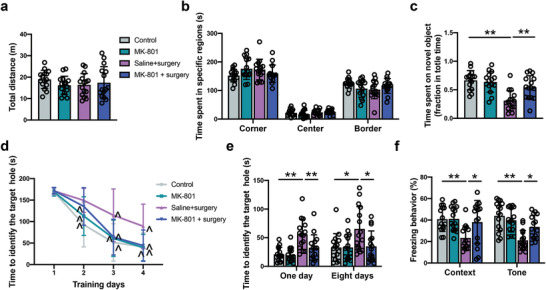
NMDA receptor inhibition by MK‐801 attenuated postoperative cognitive decline in mice. a,b) Performance in the open field test. c) Performance in the novel object recognition test. d) Performance in the training sessions of Barnes maze test. e) Performance in the memory phase of Barnes maze test. f) Performance in the context‐ and tone‐related fear conditioning test. Data are presented as mean ± S.D. with the presentation of data of each individual animal (*n* = 15). Results were analyzed by one‐way and two‐way repeated measures ANOVA (panel (d)) and one‐way ANOVA followed with Tukey test (all other panels). *P < 0.05 for the comparison, **P < 0.01 for the comparison, ^*P* < 0.05 compared with the corresponding data on day 1.

As shown above, surgery increased p‐NR1 expression/activated NMDA receptors. Inhibition of the NMDA receptors by MK‐801 in the VTA decreased the expression of p‐NR1, XBP1s, CHOP, IL‐1*β*, IL‐6, and cleaved caspase‐3 after surgery (**Figure** **8**a–d). The number of TUNEL positive staining cells in surgery mice was decreased by MK‐801 (Figure 8e). MK‐801 also maintained the number of dopaminergic neurons in the VTA after surgery (Figure 8f). Administrating MK‐801 to the VTA did not change the performance of mice in the open field test but improved the performance of surgery mice in novel object recognition test (Figure 9a–c). MK‐801 improved the performance of surgery mice in the training sessions of the Barnes maze test [F(1,28) = 13.29, *P* = 0.0011] (**Figure** **9**d). Compared to mice with surgery, mice receiving MK‐801 needed less time to identify the target hole at 1 day and 8 days after surgery (Figure 9e). MK‐801 increased the freezing behavior of surgery mice in the context‐related and tone‐related fear conditioning (Figure 9f). These results suggest that the activation of NMDA receptors in the VTA mediates surgery‐induced neuropathological changes and the impairment of learning and memory in mice.

### 2.6 Inhibition of Dopaminergic Neurons in the VTA Attenuated Surgery‐Induced Learning and Memory Dysfunction

**Figure 10:**
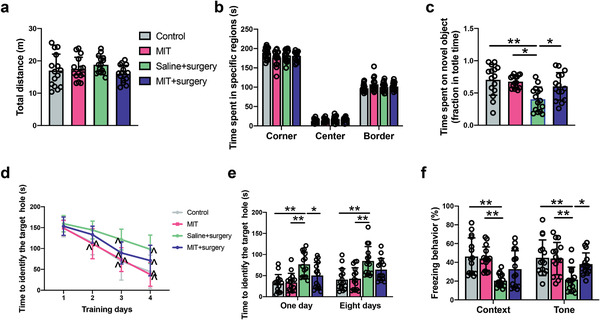
Tyrosine hydroxylase inhibition by MIT mitigated learning and memory decline in mice with surgery. a,b) Performance in the open field test. c) Performance in the novel object recognition. d) Performance in the training sessions of Barnes maze. e) Performance in the memory phase of Barnes maze. f) Performance in the context‐ and tone‐related fear conditioning test. Data are presented as mean ± S.D. with the presentation of data of each individual animal (*n* = 15). Results were analyzed by one‐way and two‐way repeated measures ANOVA (panel (d)) and one‐way ANOVA followed with Tukey test (all other panels). *P < 0.05 for the comparison, **P < 0.01 for the comparison, ^*P* < 0.05 compared with the corresponding data on day 1. MIT: 3‐Iodo‐L‐tyrosine.

To determine the role of dopaminergic neurons in the VTA in the surgical effects on learning and memory, 3‐iodo‐L‐tyrosin (MIT), a tyrosine hydroxylase inhibitor,^[^ ^33^ ^]^ was injected into the VTA. Tyrosine hydroxylase is needed for dopamine synthesis in the neurons.^[^ ^33^ ^]^ Surgery and MIT did not affect the performance of mice in the open field test (**Figure** **10**a,b). However, MIT improved the performance of surgery mice in novel object recognition test (Figure 10c). MIT also improved the performance of surgery mice in the training sessions of the Barnes maze test [F(1,28) = 16.134, *P* < 0.001] (Figure 10d). Mice with surgery and receiving MIT needed less time than mice with surgery to identify the target hole at 1 day after surgery (Figure 10e). MIT increased the freezing behavior of surgery mice in the tone‐related fear conditioning (Figure 10f). These results suggest a role of dopaminergic neurons in the VTA in surgery‐induced learning and memory dysfunction.

### 2.7 Inhibition of Endoplasmic Reticulum Stress in VTA Attenuated Surgery‐Induced Learning and Memory Dysfunction

**Figure 11:**
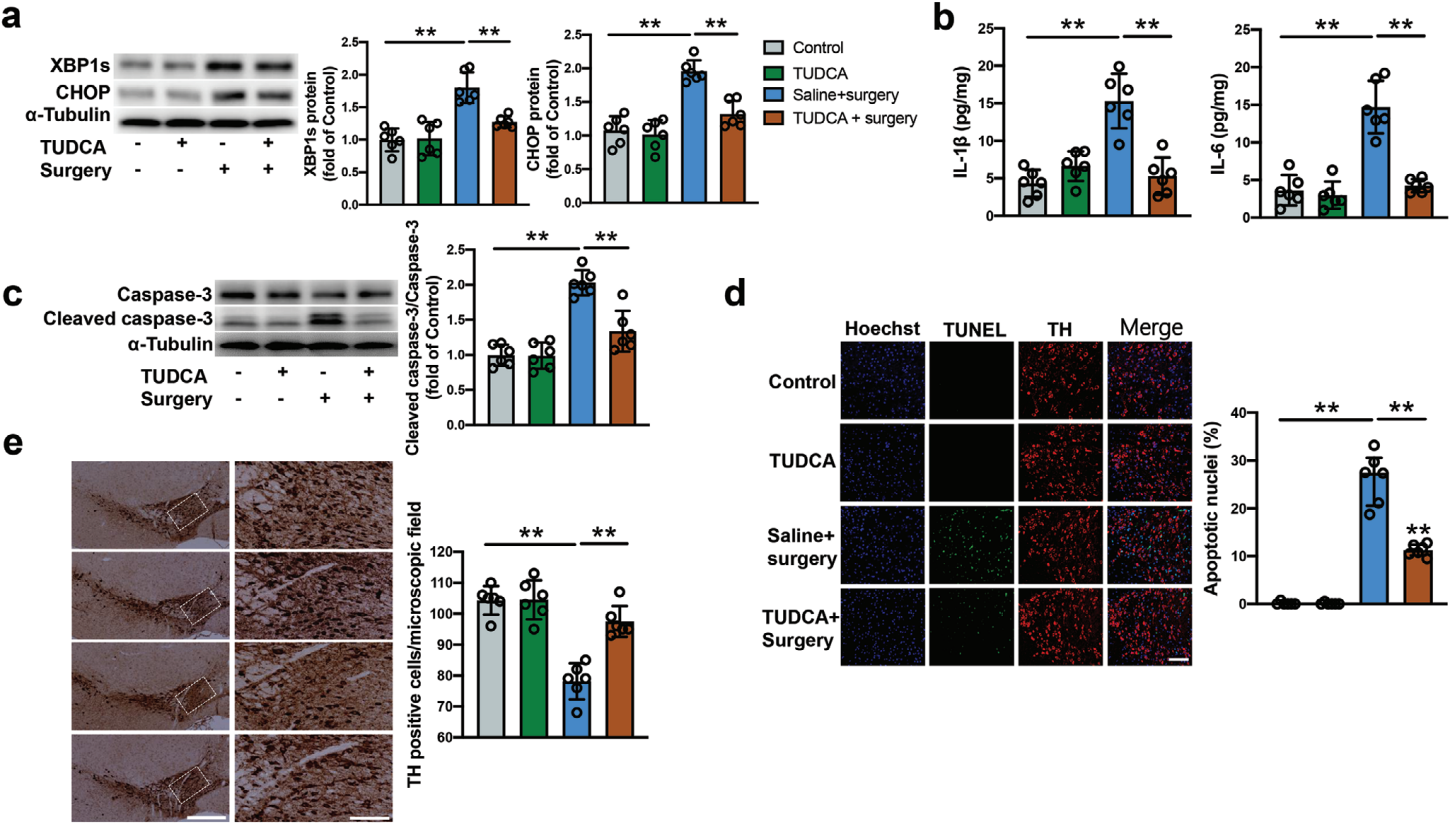
Endoplasmic reticulum stress inhibition by TUDCA reduced neuropathological changes in mice with surgery. a) Expression of XBP1s and CHOP in the VTA at 24 h after surgery. Left panel: Representative images of Western blots. Right panel: Quantitative results. b) Concentrations of IL‐1*β* and IL‐6 in the VTA at 24 h after surgery. c) Expression of caspase‐3 and cleaved caspase‐3 in the VTA at 24 h after surgery. Left panel: Representative images of Western blots. Right panel: Quantitative results. d) Number of TUNEL positive cells in the VTA at 24 h after surgery. Left panel: Representative images of TUNEL staining. Scale bar: 100 µm. Right panel: Quantitative data. e) Number of dopaminergic neurons in the VTA at 20 days after surgery. Left panel: Representative images of staining for tyrosine hydrolase (TH). Scale bar: 400 µm (left column) and 100 µm (right column). Right panel: Quantitative data of TH positive cells. Data are presented as mean ± S.D. with the presentation of data of each individual animal (*n* = 6). Results were analyzed by one‐way ANOVA followed with Tukey test. ** *P* < 0.01 for the comparison.

**Figure 12:**
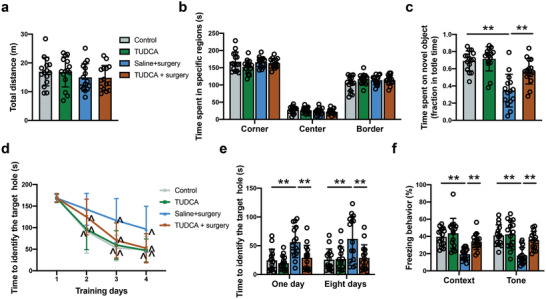
Endoplasmic reticulum stress inhibition by TUDCA attenuated learning and memory decline in mice with surgery. a,b) Performance in the open field test. c) Performance in the novel object recognition test. d) Performance in the training sessions of Barnes maze test. e) Performance in the memory phase of Barnes maze test. f) Performance in the context‐ and tone‐related fear conditioning test. Data are presented as mean ± S.D. with the presentation of data of each individual animal (*n* = 15). Results were analyzed by one‐way and two‐way repeated measures ANOVA (panel d) and one‐way ANOVA followed with Tukey test (all other panels). **P < 0.01 for the comparison, ^*P* < 0.05 compared with the corresponding data on day 1.

To determine the role of endoplasmic reticulum stress in the surgery‐induced neuropathology and POCD, an endoplasmic reticulum stress inhibitor, tauroursodeoxycholic acid (TUDCA), was injected into the VTA. As expected, inhibition of endoplasmic reticulum stress decreased the expression of XBP1s and CHOP after surgery (**Figure** **11**a). TUDCA reduced IL‐1*β* and IL‐6 concentrations and cleaved caspase‐3 expression in the VTA of surgery mice (Figure 11b,c). TUDCA also reduced the number of apoptotic cells evaluated by TUNEL staining and maintained the number of dopaminergic neurons in the VTA after surgery (Figure 11d,e). Although TUDCA did not affect the locomotor activities of surgery mice, it increased the time to explore a new object in surgery mice (**Figure** **12**a–c). TUDCA improved the performance of surgery mice in the training sessions [F(1,28) = 21.58, *P* < 0.0001] and decreased the time for these mice to identify the target hole at 1 day and 8 days after training sessions in the Barnes maze test (Figure 12d,e). TUDCA also increased the freezing behavior of surgery mice in the context‐related and tone‐related fear conditioning (Figure 12f). These results suggest that endoplasmic reticulum stress in the VTA mediates inflammatory response, cell injury and impairment of learning and memory after surgery.

## 3 Discussion

The neural circuit mechanisms for POCD, a significant syndrome during the perioperative period,^[^ ^1^, ^2^ ^]^ are not known. Our study showed that surgery activates the LHb‐VTA neural circuit and that inhibiting this circuit or injuring the LHb neurons attenuated surgery‐induced learning and memory impairment. This study represents an initial effort to identify neural circuits that are affected by surgery and provides initial evidence for the involvement of the LHb‐VTA circuit activation in the development of POCD. Interestingly, inhibiting or activating LHb by a DREADDs approach or damaging LHb by ibotenic acid did not affect the locomotor functions of mice assessed by the open field test. Similarly, inhibiting NMDA receptors, endoplasmic reticulum stress or dopamine synthesis in the VTA did not affect the locomotor functions. These results suggest that the improvement of learning and memory of surgery mice after various treatments in the LHb and VTA may not be related to the alterations of locomotor functions. In addition, there was no difference in the locomotor functions between control and surgery mice, indicating that the impairment of learning and memory caused by surgery may not be the result of locomotor dysfunction.

As we reported in a previous study,^[^ ^12^ ^]^ very few cells in the LHb expressed c‐Fos under control conditions but surgery induced a large increase in the number of c‐Fos positive cells. This increase lasted for 2 days after surgery, a time‐course that is consistent with that of surgical trauma‐induced pain and inflammation.^[^ ^34^, ^35^ ^]^ LHb is implicated in the perception of noxious and aversive stimuli^[^ ^36^ ^]^ and believed to play a vital role in regulating pain and mood^[^ ^37^ ^]^ and modulating fear memory.^[^ ^38^ ^]^ There is a direct excitatory projection from LHb to VTA and stimulating the fasciculus retroflexus fiber bundle that contains efferent projections from the LHb to the VTA^[^ ^39^, ^40^ ^]^ excites VTA dopamine neurons.^[^ ^19^ ^]^ Consistent with these previous findings, our results suggest that there are projections from LHb to VTA and that LHb may regulate VTA activity via glutamatergic signals because the tracer (mCherry) was co‐localized with vGlut2, a glutamatergic neuron biomarker,^[^ ^41^ ^]^ and inhibition of the LHb neurons reduced the level of activated NMDA receptors in the VTA. Direction inhibition or damage of the LHb or retrograde inhibition of LHb neurons via its projections to the VTA attenuated surgery‐induced learning and memory impairment. Retrograde activation of LHb neurons via its projections to the VTA induced learning and memory dysfunction. These results suggest a novel and critical role of the LHb‐VTA connections in the pathogenesis of POCD. This role may not be a surprise because LHb is known to mediate depression‐like behavior after aversive stimuli^[^ ^42^ ^]^ and surgery may be considered as an aversive stimulus.

Interestingly, LHb has projections to the RMTg that sends projections to the VTA^[^ ^22^, ^23^ ^]^ in addition to the direct projections from LHb to VTA as described above. Consistent with the existence of LHb and RMTg connections, surgery increased the number of c‐Fos positive cells in the RMTg and damage or inhibition of the LHb reduced surgery‐induced increase of c‐Fos positive cells in the RMTg. Thus, LHb neurons can regulate the activation of VTA neurons via the direct projections from LHb to VTA or via an indirect mechanism mediated by RMTg neurons in mice with surgery.

Dendritic spines are a form of microstructure for transmitting information between neurons.^[^ ^43^ ^]^ Dendritic spine density is a representative synaptic plasticity, which is closely related to learning and memory functions.^[^ ^44^, ^45^ ^]^ VTA dopaminergic neurons release dopamine to the hippocampus^[^ ^46^ ^]^ and PFC,^[^ ^47^ ^]^ which modulates memory encoding, consolidation, and retrieval.^[^ ^13^ ^]^ A previous study has shown that the dendritic spine density of the striatum changes in a dopamine concentration‐dependent manner in a Parkinson's disease animal model.^[^ ^18^ ^]^ Our previous studies have shown that the density of hippocampal dendritic spines is reduced in mice with surgery.^[^ ^31^, ^32^ ^]^ The present study found that chemogenetic suppression of the LHb reduced the VTA dopaminergic neuron damage after surgery and preserved the density of dendritic spines in the PFC and hippocampus. These results provide structural evidence for the involvement of LHb‐VTA circuit activation in POCD development.

In addition to the effect on spine density, inhibition of LHb activation or damaging LHb attenuated endoplasmic reticulum stress, inflammatory responses and cell injury in the VTA of surgery mice. These pathological processes can affect learning and memory.^[^ ^48^, ^49^ ^]^ In fact, neuroinflammation may be a major pathological process for POCD.^[^ ^50^ ^]^ Thus, endoplasmic reticulum stress, inflammatory responses and cell injury identified in this study may be general neuropathological processes for POCD.

NMDA receptors are a type of ionotropic glutamate receptors.^[^ ^51^ ^]^ NR1 is a subunit that combines with other subunits to form NMDA receptors.^[^ ^52^ ^]^ Activation of NMDA receptors induces the influx of Ca^2+^ into the cell. Ca^2+^ overloading in the cell can cause cell damage.^[^ ^53^ ^]^ The phosphorylation level of NR1 is closely related to the activity of NMDA receptors.^[^ ^54^ ^]^ In this study, the phosphorylation level of NR1 in the VTA was increased after surgery, consistent with the finding that surgery induces the activation of the LHb‐VTA circuit. Continuous activation of NMDA receptors leads to intracellular Ca^2+^ overload and induces endoplasmic reticulum stress.^[^ ^55^ ^]^ Our study showed that the injection of the NMDA receptor inhibitor MK‐801 into the VTA reduced endoplasmic reticulum stress, inflammatory response and apoptosis in the VTA and improved the cognitive functions of mice after surgery. These results clearly suggest that activation of NMDA receptors in the VTA plays a role in surgery‐induced neuropathology and dysfunction of learning and memory.

VTA is enriched with dopaminergic neurons.^[^ ^16^, ^17^, ^18^ ^]^ MIT, a tyrosine hydroxylase inhibitor^[^ ^33^ ^]^ that can inhibit dopamine synthesis, injected into the VTA attenuated surgery‐induced learning and memory impairment. These results suggest that the activation of dopaminergic neurons in the VTA plays a role in POCD.

The endoplasmic reticulum participates in the folding and post‐translational modification of almost all membrane proteins and secreted proteins.^[^ ^56^ ^]^ It plays an important role in cell homeostasis and participates in a variety of cellular functions, including protein degradation, signal transduction, lipid metabolism, and intercellular communication.^[^ ^57^ ^]^ The endoplasmic reticulum is sensitive to internal and external stimuli. Inflammation, hypoxia, oxidative stress and calcium deficiency affect the homeostasis of the endoplasmic reticulum, leading to protein folding errors and inducing endoplasmic reticulum stress.^[^ ^58^ ^]^ To restore the homeostasis of protein folding, a cell initiates a non‐folded protein response, reduces protein synthesis by increasing the phosphorylation of eukaryotic initiation factor 2*α*, and increases the folding and degradability of endoplasmic reticulum proteins through the transcriptional activation of XBP1 and activating transcription factor 6*α*.^[^ ^59^ ^]^ If the unfolded protein response fails to restore the homeostasis of the endoplasmic reticulum, apoptosis occurs because of the activation of the following pathways: endoplasmic reticulum stress induces continuous release of Ca^2+^, which causes cytochrome c to bind apoptotic peptidase activating factor 1 in the cytoplasm to activate caspase 3.^[^ ^60^ ^]^ Under normal physiological conditions, CHOP is expressed at a very low level. The phosphorylation of eukaryotic initiation factor 2*α* and the transcriptional activation of activating transcription factor 6*α* caused by endoplasmic reticulum stress up‐regulate the transcription of the pro‐apoptotic gene *chop*, leading to cell apoptosis.^[^ ^61^ ^]^ In this study, the level of XBP1s and CHOP in the surgery mice was reduced by the endoplasmic reticulum stress inhibitor TUDCA injected into the VTA. TUDCA also reduced inflammation and apoptosis and improved the cognitive functions of mice after surgery. These data suggest a role of endoplasmic reticulum stress in the development of POCD.

Our findings suggest that the activation of the LHb‐VTA neural circuit in mice with surgery is necessary and sufficient for POCD. This activation involves glutamate receptors to activate dopaminergic neurons and induce endoplasmic reticulum stress and inflammation in the VTA, which ultimately leads to a decreased number of neurons and impaired dendritic spines at a delayed phase. These cascade events may be the mechanisms for the presentation of POCD at acute and delayed phases.^[^ ^31^, ^49^, ^62^ ^]^ Of note, a lower volume of the temporal lobe is associated with the occurrence of POCD in humans.^[^ ^63^ ^]^ However, there is no clinical study defining neural circuit activation or inhibition in POCD.

Three paradigms were used to measure learning and memory for the consideration that multiple domains of cognition may be affected in patients with POCD. Novel object recognition requires the functions of perirhinal cortex and hippocampus.^[^ ^64^ ^]^ Barnes maze tests spatial learning and memory and needs the functions of hippocampus, entorhinal cortex and other cortical areas.^[^ ^65^, ^66^ ^]^ The performance of fear conditioning depends on amygdala, cerebral cortex, and hippocampus.^[^ ^67^ ^]^ Thus, functions of many brain regions, in addition to those of hippocampus, were tested in our study.

Our study has limitations. There was a decrease in the dendritic spine density in the PFC and hippocampus and a reduction of dopaminergic neurons in the VTA after surgery. VTA has a large number of dopaminergic neurons that send projections to the PFC and hippocampus.^[^ ^16^, ^17^, ^18^, ^30^ ^]^ One will infer that dopaminergic neurons in the VTA are involved in the development of POCD. This inference is supported by our finding that MIT attenuated POCD. However, additional evidence is needed to exclude a role of non‐dopaminergic neurons of the VTA in POCD. Also, the phosphorylation level of NR1 was used to indicate the activation of NMDA receptors. This activation was not confirmed with electrophysiological measurements. However, the phosphorylation of NR1 was decreased by MK‐801 in our study, suggesting the appropriate use of NR1 phosphorylation as an indicator for NMDA receptor activation. Finally, our study was performed in male mice. Studies using female mice are needed to determine whether there is a sex‐difference in the findings presented here.

**Figure 13:**
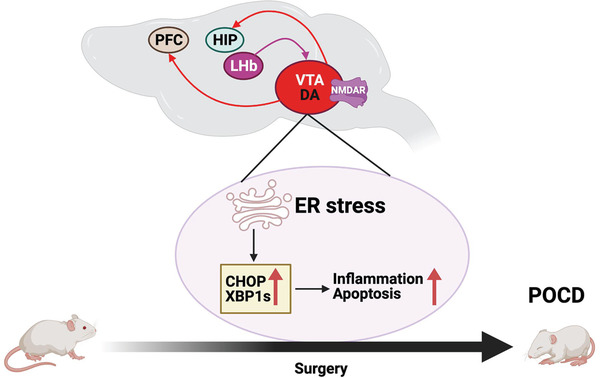
Diagram of possible neural circuits and molecules for postoperative cognitive dysfunction. PFC: prefrontal cortex, HIP: hippocampus, LHb: Lateral habenula, CHOP: CCAAT‐enhancer‐binding protein homologous protein, DA: Dopamine, ER: endoplasmic reticulum, NMDAR: *N*‐methyl‐d‐aspartate receptors, POCD: postoperative cognitive dysfunction, VTA: ventral tegmental area, XBP1s: X‐box binding protein 1.

In conclusion, our results suggest novel neuronal circuit and molecular mechanisms that mediate the development of POCD: surgery activates the LHb neurons including the glutamatergic neurons in this brain region, which leads to endoplasmic reticulum stress, inflammatory responses and dopaminergic neuronal injury in the VTA that results in a decrease of dendritic spine density in the PFC and hippocampus (**Figure** **13**).

## 4 Experimental Section

The protocol of animal experiments in this study was approved by the Institutional Animal Care and Use Committee at the University of Virginia (Charlottesville, VA, USA) (protocol number: 3114, to Zhiyi Zuo's laboratory). All animal experiments were conducted in accordance with the National Institutes of Health Guide for the Care and Use of Laboratory Animals (NIH publication number 80‐23, revised in 2011).

### Animals and Experimental Groups

Six‐ to eight‐week‐old CD‐1 male mice, weighing 31–36 g, were purchased from Charles River Laboratory Inc. (Wilmington, MA), and maintained under a 12 h light/dark cycle with free access to food and water.

In experiment 1 (Figure S5a, Supporting Information), mice were randomly assigned to a control group or a surgery group.

In experiment 2 (Figure S5b, Supporting Information), five cohorts of mice and the DREADDs technique were used to test the role of the LHb‐VTA circuit in POCD. AAV2‐hSyn‐mCherry (5 × 10¹^2^ vg mL^−1^) 200 nL per side was injected into the LHb to determine the neural projection from LHb to VTA in the first set of experiments. In the second set of experiments, the retrograde virus AAV2‐retro‐CAG‐GFP (≥ 7 × 10¹^2^ vg mL^−1^) 300 nL was injected into the VTA. Four weeks later, these mice were subjected to carotid artery exposure. Mouse brains were harvested 3 h after surgery and frozen sections were used to examine the projections of LHb, PFC or hippocampus to VTA. The DREADDs technique was used in the following three sets of experiments to inhibit the LHb‐VTA circuit after surgery. First, mice were randomly assigned to four groups: control, surgery plus hM4Di plus saline, surgery plus mCherry plus compound 21, and surgery plus hM4Di plus compound 21. Mice were bilaterally injected with AAV2‐hsyn‐hM4Di‐mCherry (7 × 10¹^2^ vg mL^−1^), AAV2‐hsyn‐mCherry (5 × 10¹^2^ vg mL^−1^) or normal saline 200 nL per side into the LHb four weeks before surgery. The DREADDs agonist compound 21 was used to activate hM4Di^[^ ^12^ ^]^ to inhibit the activation of LHb. Compound 21 was dissolved in normal saline and was injected intraperitoneally with the first dose at 30 min before surgery, 3 times a day, until 2 days after the operation. Second, to determine the role of the LHb neuronal projections to the VTA in POCD, mice were randomly assigned to the following five groups: control, surgery, surgery plus hM4Di plus saline, surgery plus mCherry plus compound 21, and surgery plus hM4Di plus compound 21. Mice were bilaterally injected with AAV2/2 retro plus‐hSyn‐Cre‐WPRE‐pA (1 × 10^13^ vg mL^−1^) 300 nL per side into the VTA and AAV2/9‐hSyn‐DIO‐hM4Di‐mCherry‐WPRE‐pA (1 × 10^13^ vg mL^−1^) or AAV2/9‐hSyn‐DIO‐mCherry‐WPRE‐pA (1 × 10^13^ vg mL^−1^) 200 nL per side into the LHb four weeks before surgery. Compound 21 or the same volume of saline was injected intraperitoneally 30 min before surgery, 3 times a day, until 2 days after the operation. Third, to determine the effect of activating the LHb‐VTA circuit on cognitive performance, mice were randomly assigned to the following four groups: control, hM3Dq plus saline, mCherry plus compound 21, and hM3Dq plus compound 21. Mice were bilaterally injected with AAV2/2 retro plus‐hSyn‐Cre‐WPRE‐pA (1 × 10^13^ vg mL^−1^) 300 nL per side into the VTA and AAV2/9‐hSyn‐DIO‐hM3Dq‐mCherry‐WPRE‐pA (1 × 10^13^ vg mL^−1^) or AAV2/9‐hSyn‐DIO‐mCherry‐WPRE‐pA (1 × 10^13^ vg mL^−1^) 200 nL per side into the LHb four weeks before behavioral tests. Compound 21 or the same volume of saline was injected intraperitoneally 3 times a day for 3 days (day 0 to day 2). Animals were subjected to the behavioral tests from day 4.

In experiment 3 (Figure S5c, Supporting Information), induction of LHb lesion was used to supplement the DREADDs approach. Mice were randomly assigned to four groups: control, ibotenic acid lesion, saline plus surgery, and ibotenic acid lesion plus surgery. Mice were bilaterally injected with ibotenic acid or normal saline into the LHb 14 days before surgery.

To determine the role of NMDA receptors in mediating POCD via the activation of the LHb‐VTA circuit, four groups of mice were included in experiment 4 (Figure S5d, Supporting Information): control, MK‐801, saline plus surgery, and MK‐801 plus surgery. MK‐801 was dissolved in normal saline and bilaterally injected into the VTA 30 min before surgery and then once every day for the first two days after surgery. The control and saline plus surgery groups received injections of normal saline at a volume equal to that of MK‐801 solution.

To determine the role of dopaminergic neurons in mediating the cognitive dysfunction after surgery, mice were randomly assigned to the following four groups in experiment 5 (Figure S5e, Supporting Information): control, MIT, saline plus surgery, and MIT plus surgery. MIT was dissolved in saline and bilaterally injected into the VTA 30 min before surgery and the first two days after surgery. Equal volume of normal saline to that of MIT was injected into the VTA of control group and saline plus surgery group.

Experiment 6 (Figure S5f, Supporting Information) was conducted to investigate the involvement of endoplasmic reticulum stress in the effects of LHb‐VTA circuit on POCD. The following four groups were included: control, TUDCA, saline plus surgery, and TUDCA plus surgery. TUDCA, an endoplasmic reticulum stress inhibitor,^[^ ^68^ ^]^ was dissolved in normal saline and bilaterally injected into the VTA 30 min before surgery and then once every day for the first two days after surgery. Equal volume of normal saline to that of TUDCA was injected into the VTA of control group and saline plus surgery group.

To determine the diffusion of solution after being injected, 0.1% Evans blue was injected into the LHb (200 nL per side) and VTA (300 nL per side). The brain was harvested 3 h after the injection and 100 µm‐thick sections were cut and observed under a microscope.

### Animal Surgery

The right carotid artery exposure surgery was used as the model to induce POCD.^[^ ^50^ ^]^ Briefly, mice were anesthetized by 1.8% isoflurane whose concentration was monitored with a DatexTM infrared analyzer (Capnomac, Helsinki, Finland). Their temperature was maintained by a warm pad. The mouse was placed in a supine position and a 1.5‐cm midline neck incision was made after the mouse was exposed to isoflurane for at least 30 min. The soft tissues over the trachea were retracted gently. One centimeter long right common carotid artery was dissected carefully free from adjacent tissues without damaging vagus nerve. The wound was then irrigated and closed by using surgical suture. The surgical procedure was performed under sterile conditions and lasted around 15 min. After the surgery, all animals received a subcutaneous injection of 3 mg kg^−1^ bupivacaine for postoperative analgesia. After a total of 2‐h anesthesia, the mouse was allowed to wake up spontaneously.

### Virus or Chemical Injection

The viruses AAV2‐hSyn‐hM4D(Gi)‐mCherry (Addgene, catalogue number: 50 475), AAV2‐hSyn‐mCherry (Addgene, catalogue number: 114 472), AAV2/9‐hSyn‐DIO‐hM4Di‐mCherry‐WPRE‐pA (Tailtool Bioscience, Shanghai, China; catalogue number: S0193‐9) or AAV2/9‐hSyn‐DIO‐mCherry‐WPRE‐pA (Tailtool Bioscience, catalogue number: S0240‐9), or AAV2/9‐hSyn‐DIO‐hM3Dq‐mCherry‐WPRE‐pA (Tailtool Bioscience, catalogue number: S0192‐9) were stereotaxically injected bilaterally into the LHb (coordinates: anteroposterior −1.7 mm from the bregma, mediolateral ±0.6 mm, dorsoventral −2.7 mm from dura) using a 1 µL Hamilton Neuros syringe 7001 KH at a volume of 200 nL per side and a rate of 25 nL min^−1^. Similarly, AAV2/2 retro plus‐hSyn‐Cre‐WPRE‐pA (Tailtool Bioscience, catalogue number: S0278‐2RP) or AAV2‐retro‐CAG‐GFP (Addgene, catalogue number: 37825‐AAVrg) was stereotaxically injected bilaterally into the VTA (coordinates: anteroposterior −2.3 mm from the bregma, mediolateral ±0.5 mm, dorsoventral −4.5 mm from dura) at a volume of 300 nL per side and a rate of 50 nL min^−1^. Mice were returned to their cages for 4 weeks. Ibotenic acid (Millipore, catalogue number: 5.05024.0001), a neurotoxin,^[^ ^24^ ^]^ was dissolved in normal saline to a concentration of 1 mg ml^−1^ and bilaterally injected into the LHb at a volume of 100 nL per side. Mice were used 2 weeks after the LHb lesion. The NMDA receptor antagonist MK‐801 (500 µg mL^−1^)^[^ ^69^ ^]^ (Sigma‐Aldrich, catalogue number: M107), the tyrosine hydroxylase inhibitor MIT (3 µg mL^−1^)^[^ ^33^ ^]^ (APExBIO, catalogue number: C7043) or the endoplasmic reticulum stress antagonist TUDCA (100 µg mL^−1^)^[^ ^68^ ^]^ (Millipore, catalogue number: 580 549) was stereotaxically injected bilaterally into the VTA at a volume of 300 nL per side and a rate of 50 nL min^−1^. After the injection, mice were allowed to wake up from the anesthesia. Mice were anesthetized again 30 min later and underwent the right carotid artery exposure surgery. MK‐801, MIT and TUDCA were injected once a day for two consecutive days after surgery. Compound 21 (C21) (Hello Bio, catalogue number: HB6124) was dissolved in normal saline to a concentration of 1 mg mL^−1^ and injected 1 mg kg^−1^ intraperitoneally 30 min before right carotid artery exposure surgery, 3 times a day, until 2 days after the operation.

### Behavioral Tests

Open field test was used to assess locomotor activities of mice. The novel object recognition, Barnes maze and fear conditioning tests were used to determine learning and memory functions.

Open field test was performed on the 4th day after surgery. As described before,^[^ ^70^ ^]^ mice were placed into the open field box for 5 min. The ANY‐maze behavioral tracking software (SD Instruments) was used to record the distance traveled and time spent in the corner, border and center areas of the open field.

One day after open field test, mice were subjected to novel object recognition test. As we described previously,^[^ ^70^ ^]^ two identical objects were placed in opposite sides of the box during a training session. The mouse was placed in the middle of the box and allowed to explore for 5 min. One hour later, one of the training objects was replaced with a novel object. The mouse was allowed to explore for 5 min. The ANY‐maze software was used to track and record the time exploring objects. The ratio of time spent on the new object to total exploration time on both objects was calculated.

Mice were subjected to the Barnes maze test on the 6th day after surgery to assess spatial learning and memory. As we did before,^[^ ^50^ ^]^ the mouse was placed in the center of a circular platform with 20 equally spaced holes. One of the holes was connected to a dark chamber called target hole (SD Instruments). Aversive noise (85 dB) and bright light (200 W) shed on the platform were used to encourage the mouse to find the target hole. The spatial acquisition training phase was 4 days with 2 trials per day, 3 min per trial, and 2 h interval between each trial. The short‐term and long‐term spatial memory tests were performed on the first and eighth day after training phase. No test was performed during the period from first to eighth day after training phase. The latency to find the target hole was recorded by the ANY‐maze video tracking system.

A fear conditioning test was conducted 24 h after the Barnes maze test to measure the associative memory of mice. As we previously described,^[^ ^50^ ^]^ the mouse was placed into a test chamber wiped with 70% alcohol and exposed to 3 tone‐foot shock pairings (tone: 2000 Hz, 85db, 30 s; foot shock: 0.7 mA, 2 s) with an inter‐pairing interval of 1 min in a relatively dark room. The mouse was removed from the test chamber 30 s after the conditioning stimuli. Mice were placed back into the same test chamber for 6 min but without tone and shock 24 h after being exposed to the conditioning stimuli. The animal was placed into another test chamber that had different context and smell and wiped with 1% acetic acid in a relatively light room 2 h later. Freezing behavior was recorded for 3 min without the tone stimulus. The tone was then turned on for 3 cycles, each cycle for 30 s followed by 1‐min inter‐cycle interval (a total of 4.5 min). The length of context‐related freezing behavior during the 6 min in the first chamber and tone‐related freezing behavior during the 4.5 min in the second chamber was recorded by an observer who was blind to the group assignment.

### Brain Tissue Collection

Mice were deeply anesthetized with isoflurane and transcardially perfused with 4% paraformaldehyde 4 weeks after viral injection, 2 weeks after ibotenic acid lesion, and 3 h, 24 h, and 20 days after surgery. The coronal brain slices from bregma −1.5 to −2.1 mm or −2.9 to −3.8 mm were used for immunofluorescent, immunohistochemistry, TUNEL or Nissl staining of the LHb and VTA, respectively.

Brain tissues in another set of experiments were immediately removed and the LHb and VTA were quickly dissected out on ice at 3, 24, 48 and 72 h after surgery for Western blotting or enzyme‐linked immunosorbent assay (ELISA) analysis.

Mouse brain was quickly removed 20 days after surgery and immerged in the AB mixture for Golgi staining.

### Immunofluorescent Staining

Neuronal activation was assessed by detecting the expression of c‐Fos using immunofluorescent staining as we previously described.^[^ ^12^ ^]^ Briefly, brains were fixed in 4% paraformaldehyde for 3 days at 4 °C and then embedded in paraffin. Coronal 5‐µm thick brain sections at −1.5 to −2.1 mm (for LHb) or −2.9 to −3.8 mm (for VTA) from bregma were cut and mounted on slides. Antigen retrieval was performed in sodium citrate buffer (10 × 10^−3^ m sodium citrate, 0.05% Tween 20, pH 6.0) at 95–100 °C for 20 min. After being blocked in 5% donkey serum plus 1% bovine serum albumin in Tris‐buffered saline (TBS) for 2 h at room temperature, sections were incubated with the primary antibodies mouse anti‐c‐Fos monoclonal antibody (1:1000, Abcam, catalogue number: ab208942), rabbit polyclonal anti‐c‐Fos (1:1000, Abcam, catalogue number: ab190289), mouse monoclonal anti‐NeuN (1:1000, Sigma‐Aldrich, catalogue number: MAB377) or sheep anti‐tyrosine hydroxylase (TH) polyclonal antibody (1:1000, Invitrogen, catalogue number: PA1‐4679) at 4 °C overnight. Sections were washed with Tris‐buffered saline (TBS), and incubated with the secondary antibodies donkey anti‐mouse IgG antibody conjugated with Alexa Fluor 488 (1:500, Invitrogen, catalogue number: A21202), donkey anti‐rabbit IgG antibody conjugated with Alexa Fluor 594 (1:200, Invitrogen, catalogue number: A21207) or donkey anti‐sheep IgG antibody conjugated with Alexa Fluor 647 (1:500, Abcam, catalogue number: ab150179) at room temperature in the dark for 1 h. Cell nuclei were stained by Hoechst 33 342 (1:1000, Thermo Scientific, catalogue number: 62 249) at room temperature for 15 min in the dark. Images were acquired with a confocal microscope (Zeiss 710) and three sections per mouse were analyzed. The results of the three sections were averaged to reflect the expression level of a protein in the mouse.

To determine cell types that expressed mCherry whose code was carried by the viral vector, 25‐µm thick frozen coronal sections containing the LHb were cut and incubated with rabbit anti‐NeuN monoclonal antibody (1:1000, Abcam, catalogue number: ab177487) and then donkey anti‐rabbit IgG antibody conjugated with Alexa Fluor 488 (1:500, Invitrogen, catalogue number: A31573) for detecting NeuN expression. Sections in the VTA were incubated with mouse anti‐VGluT2 monoclonal antibody (1:1000, Abcam, catalogue number: ab79157) followed by donkey anti‐mouse IgG antibody conjugated with Alexa Fluor 488 (1:500, Invitrogen, catalogue number: A21202) or rabbit anti‐VGAT polyclonal antibody (1:1000, Abcam, catalogue number: ab235952) detected by donkey anti‐rabbit IgG antibody conjugated with Alexa Fluor 488 (1:500, Invitrogen, catalogue number: A21207).

### Nissl Staining

The LHb lesion by ibotenic acid was confirmed by Nissl staining as we did before.^[^ ^12^ ^]^ After being deparaffinized and rehydrated, sections were stained with 0.1% crystal violet solution at 37°C for 10 min. Sections were rinsed quickly in distilled water, differentiated in alcohol, cleaned in xylene and mounted with permanent mounting medium (Fisher Scientific, catalogue number: 192 497). Images were acquired with a bright field microscope (Olympus DP70).

### Western Blotting

Briefly, the LHb and VTA were collected on ice and homogenized in RIPA buffer (Thermo Scientific, catalogue number: 89 901) with protease inhibitor cocktail (Sigma, catalogue number: P2714) and phosphatase inhibitor cocktail (Roche, catalogue number: 0 469 313 2001). Protein concentration was determined using Pierce BCA Protein Assay Kit (Thermo Scientific, catalogue number: 23 228). Equal amounts of proteins were loaded onto 4–20% SDS‐PAGE gels (Bio‐Rad, catalogue number: 4 568 094). Proteins were transferred to a polyvinylidene difluoride membrane (Bio‐Rad, catalogue number: 1 620 177) and blocked with a blocking buffer (Thermo Scientific, catalogue number: 37 573) at room temperature for 1 h. The membrane was incubated with the primary antibodies anti‐c‐Fos monoclonal antibody (1:1000, Abcam, catalogue number:ab208942), anti‐NR1 monoclonal antibody (1:1000, Invitrogen, catalogue number: 320 500), anti‐p‐NR1 polyclonal antibody (1:1000, Millipore, catalogue number: ABN99), anti‐XBP1 monoclonal antibody (1:1000, Cell Signaling Technology, catalogue number: 12782s), anti‐CHOP monoclonal antibody (1:1000, Cell Signaling Technology, catalogue number: 2895s), anti‐caspase‐3 polyclonal antibody (1:1000, Cell Signaling Technology, catalogue number: 9662s), anti‐cleaved Caspase‐3 polyclonal antibody (1:1000, Cell Signaling Technology, catalogue number: 9661s) or anti‐*α*‐tubulin monoclonal antibody (1:1000, Cell Signaling Technology, catalogue number: 5335s) overnight at 4 °C. The secondary antibodies goat anti‐rabbit IgG antibody or goat anti‐mouse IgG antibody conjugated with horseradish peroxidase (1:5000, Santa Cruz Biotechnology) were used. The protein bands were detected by enhanced chemiluminescence and quantified by Genetools version 4.01. The amount of phosphorylated proteins was normalized by the total amount of these proteins. Cleaved caspase 3 was normalized by total caspase 3. Other proteins were normalized by *α*‐tubulin. The results of various experimental conditions were then normalized by the results of corresponding control group.

### Enzyme‐Linked Immunosorbent Assay

Cytokines were detected as we did before.^[^ ^71^ ^]^ The mice were anesthetized with isoflurane 24 h after surgery and their VTA was dissected out on ice and homogenized in RIPA buffer (Thermo Scientific, catalogue number: 89 901) with protease inhibitor cocktail (Sigma, catalogue number: P2714). IL‐1*β* and IL‐6 were quantified using ELISA kits following the manufacturer's instructions (R&D Systems). The concentrations of IL‐1*β* and IL‐6 in the VTA were standardized to the protein contents.

### TUNEL Staining

As we did before,^[^ ^71^ ^]^ TUNEL staining was performed to detect apoptotic cells at 24 h after surgery in the VTA using an *in‐situ* apoptosis detection kit (Millipore, catalogue number: S7110) in accordance with the manufacturer's protocol. Sections were co‐stained with an anti‐TH polyclonal antibody (1:1000, Invitrogen, catalogue number: PA1‐4679) and donkey anti‐sheep IgG antibody conjugated with Alexa Fluor 647 (1:500, Abcam, catalogue number: ab150179). Images were captured using a confocal microscope (Zeiss 710). The percentage of nuclei marked with green fluorescence (TUNEL positive staining) in all nuclei labeled with Hoechst 33 342 was calculated. Three sections per mouse were analyzed.

### Immunohistochemistry Staining

Coronal 5‐µm thick brain sections harvested 20 days after surgery were processed for immunohistochemical staining to count dopaminergic neurons as previously described.^[^ ^72^ ^]^ Briefly, sections were deparaffinized, antigen retrieved, and endogenous peroxidase activity inactivated. Dopaminergic neurons were identified using an anti‐TH polyclonal antibody (1:1000, Invitrogen, catalogue number: PA1‐4679) and goat anti‐sheep IgG antibody conjugated with horseradish peroxidase (1:500, Santa Cruz Biotechnology, catalogue number: C0513). Immunoreactivity was detected with a bright‐field microscope (Olympus DP70) and analyzed by Image J software. Three sections per mouse were analyzed.

### Golgi Staining

The prefrontal cortical and hippocampal dendritic spines were detected using the FD Rapid Golgi Stain^TM^ Kit (FD Neurotech, catalogue number: PK401) as we did before.^[^ ^31^ ^]^ Brains were harvested 20 days after surgery and incubated in impregnation solutions for 2 weeks at room temperature in the dark. The brains were transferred to solution C for 1 week and 150‐µm thick coronal sections at around 1.9 mm and −2.7 mm from bregma were cut on a vibratome (Ted Pella 10 110). The tissues were mounted to gelatin‐coated slides (FD Neurotech, catalogue number: 101 281) and stained. Images were taken by using a Zeiss Axio Imager Z2 microscope. Neurons in the PFC and hippocampus were randomly selected from each mouse and image series of z‐stack were taken at intervals of 0.1 µm with a ×100 oil objective and intervals of 1.0 µm with a ×40 oil objective. The MBF software (MBF Bioscience) was used for dimensional reconstruction. Five neurons were randomly selected from each animal for quantitative analysis.

### Statistical Analysis

All data were analyzed by GraphPad Prism 8.0. The protein expression data as detected by Western blotting were normalized as described in section 4.8. Since there was no plan to exclude any data, evaluation of outliers was not performed. The Shapiro‐Wilk test was used to test the normal distribution of the data and all data are present as mean ± S.D. with the presentation of data of each individual animal in the bar graphs. The sample size for each experiment was described in the figure legends. The results were analyzed by using Student's *t* test, one‐way or two‐way analysis of variance (ANOVA) followed with Tukey test as appropriate. Two‐way or one‐way repeated measures ANOVA was used to compare the data of Barnes maze training sessions between groups and within one group, respectively. Differences were considered significant at a *P* < 0.05 based on two‐tailed hypothesis testing.

## Conflict of Interest

The authors declare no conflict of interest.

## Author Contributions

Z.Z. conceived the project. J.X., W.S., J.L., H.Y., and Z.Z. designed the studies. J.X. and W.S. performed the experiments and initial data analysis. J.X. drafted the manuscript. Z.Z. revised the manuscript.

## Supporting information

Supporting Information

## Data Availability

The data that support the findings of this study are available from the corresponding author upon reasonable request.

## References

[1] T. G. Monk , B. C. Weldon , C. W. Garvan , D. E. Dede , M. T. van der Aa , K. M. Heilman , J. S. Gravenstein , Anesthesiology 2008, 108, 18. 18156878 10.1097/01.anes.0000296071.19434.1e

[2] M. Berger , K. J. Schenning , C. H. Brown , S. G. Deiner , R. A. Whittington , R. G. Eckenhoff , M. S. Angst , S. Avramescu , A. Bekker , M. Brzezinski , G. Crosby , D. J. Culley , M. Eckenhoff , L. I. Eriksson , L. Evered , J. Ibinson , R. P. Kline , A. Kofke , D. Ma , J. P. Mathew , M. Maze , B. A. Orser , C. C. Price , D. A. Scott , B. Silbert , D. Su , N. Terrando , D. S. Wang , H. Wei , Z. Xie , Anesth. Analg. 2018, 127, 1406. 30303868 10.1213/ANE.0000000000003841 PMC6309612

[3] J. Steinmetz , K. B. Christensen , T. Lund , N. Lohse , L. S. Rasmussen , Anesthesiology 2009, 110, 548. 19225398 10.1097/ALN.0b013e318195b569

[4] I. B. Hovens , R. G. Schoemaker , E. A. van der Zee , E. Heineman , G. J. Izaks , B. L. van Leeuwen , Brain, Behav., Immun. 2012, 26, 1169. 22728316 10.1016/j.bbi.2012.06.004

[5] R. G. Eckenhoff , M. Maze , Z. Xie , D. J. Culley , S. J. Goodlin , Z. Zuo , H. Wei , R. A. Whittington , N. Terrando , B. A. Orser , M. F. Eckenhoff , Anesthesiology 2020, 132, 55. 31834869 10.1097/ALN.0000000000002956 PMC6913778

[6] I. H. Bianco , S. W. Wilson , Philos. Trans. R. Soc. London, Ser. B 2009, 364, 1005. 19064356 10.1098/rstb.2008.0213 PMC2666075

[7] L. Lecourtier , P. H. Kelly , Neurosci. Biobehav. Rev. 2007, 31, 658. 17379307 10.1016/j.neubiorev.2007.01.004

[8] S. Geisler , M. Trimble , CNS Spectrums 2008, 13, 484. 18567972 10.1017/s1092852900016710

[9] O. Hikosaka , Nat. Rev. Neurosci. 2010, 11, 503. 20559337 10.1038/nrn2866 PMC3447364

[10] R. Goutagny , M. Loureiro , J. Jackson , J. Chaumont , S. Williams , P. Isope , C. Kelche , J. C. Cassel , L. Lecourtier , Neuropsychopharmacology 2013, 38, 2418. 23736315 10.1038/npp.2013.142 PMC3799061

[11] H. Aizawa , S. Yanagihara , M. Kobayashi , K. Niisato , T. Takekawa , R. Harukuni , T. J. McHugh , T. Fukai , Y. Isomura , H. Okamoto , J. Neurosci. 2013, 33, 8909. 23678132 10.1523/JNEUROSCI.4369-12.2013 PMC6618841

[12] Q. Zeng , W. Shan , H. Zhang , J. Yang , Z. Zuo , Theranostics 2021, 11, 3813. 33664863 10.7150/thno.45690 PMC7914349

[13] B. Engelhard , J. Finkelstein , J. Cox , W. Fleming , H. J. Jang , S. Ornelas , S. A. Koay , S. Y. Thiberge , N. D. Daw , D. W. Tank , I. B. Witten , Nature 2019, 570, 509. 31142844 10.1038/s41586-019-1261-9 PMC7147811

[14] L. S. Zweifel , J. G. Parker , C. J. Lobb , A. Rainwater , V. Z. Wall , J. P. Fadok , M. Darvas , M. J. Kim , S. J. Mizumori , C. A. Paladini , P. E. Phillips , R. D. Palmiter , Proc. Natl. Acad. Sci. USA 2009, 106, 7281. 19342487 10.1073/pnas.0813415106 PMC2678650

[15] A. Nieoullon , Prog. Neurobiol. 2002, 67, 53. 12126656 10.1016/s0301-0082(02)00011-4

[16] A. Bjorklund , S. B. Dunnett , Trends Neurosci. 2007, 30, 194. 17408759 10.1016/j.tins.2007.03.006

[17] J. I. Broussard , K. Yang , A. T. Levine , T. Tsetsenis , D. Jenson , F. Cao , I. Garcia , B. R. Arenkiel , F. M. Zhou , M. De Biasi , J. A. Dani , Cell Rep. 2016, 14, 1930. 26904943 10.1016/j.celrep.2016.01.070 PMC4772154

[18] S. Alberquilla , A. Gonzalez‐Granillo , E. D. Martin , R. Moratalla , Neurobiol. Dis. 2020, 134, 104666. 31682992 10.1016/j.nbd.2019.104666

[19] P. L. Brown , P. D. Shepard , J. Neurophysiol. 2016, 116, 1161. 27358317 10.1152/jn.00305.2016 PMC5013172

[20] H. Ji , P. D. Shepard , J. Neurosci. 2007, 27, 6923. 17596440 10.1523/JNEUROSCI.0958-07.2007 PMC6672239

[21] M. Matsumoto , O. Hikosaka , Nature 2007, 447, 1111. 17522629 10.1038/nature05860

[22] H. Li , D. Pullmann , J. Y. Cho , M. Eid , T. C. Jhou , Elife 2019, 8, 41542. 10.7554/eLife.41542 PMC6361585 30667358

[23] P. M. Baker , W. Bair , J. Neurosci. 2016, 36, 6563. 27307243 10.1523/JNEUROSCI.3213-15.2016 PMC6601923

[24] O. Isacson , P. Brundin , P. A. Kelly , F. H. Gage , A. Bjorklund , Nature 1984, 311, 458. 6482962 10.1038/311458a0

[25] H. Aizawa , M. Kobayashi , S. Tanaka , T. Fukai , H. Okamoto , J. Comp. Neurol. 2012, 520, 4051. 22700183 10.1002/cne.23167

[26] K. B. Hansen , F. Yi , R. E. Perszyk , H. Furukawa , L. P. Wollmuth , A. J. Gibb , S. F. Traynelis , J. Gen. Physiol. 2018, 150, 1081. 30037851 10.1085/jgp.201812032 PMC6080888

[27] X. Zhou , D. Hollern , J. Liao , E. Andrechek , H. Wang , Cell Death Dis. 2013, 4, 560. 10.1038/cddis.2013.82 PMC3615746 23538441

[28] F. J. Guo , Y. Liu , J. Zhou , S. Luo , W. Zhao , X. Li , C. Liu , Histochem. Cell Biol. 2012, 138, 447. 22669460 10.1007/s00418-012-0967-7

[29] S. Oyadomari , M. Mori , Cell Death Differ. 2004, 11, 381. 14685163 10.1038/sj.cdd.4401373

[30] K. T. Beier , E. E. Steinberg , K. E. DeLoach , S. Xie , K. Miyamichi , L. Schwarz , X. J. Gao , E. J. Kremer , R. C. Malenka , L. Luo , Cell 2015, 162, 622. 26232228 10.1016/j.cell.2015.07.015 PMC4522312

[31] F. Luo , J. Min , J. Wu , Z. Zuo , Mol. Neurobiol. 2020, 57, 3702. 32564283 10.1007/s12035-020-01987-2 PMC7415726

[32] J. Zhong , J. Li , C. Ni , Z. Zuo , Front. Aging Neurosci. 2020, 12, 605330. 33324197 10.3389/fnagi.2020.605330 PMC7726433

[33] P. F. Fitzpatrick , Biochemistry 1991, 30, 3658. 1673058 10.1021/bi00229a010

[34] E. M. Pogatzki‐Zahn , D. Segelcke , S. A. Schug , Pain Rep. 2017, 2, 588. 10.1097/PR9.0000000000000588 PMC5770176 29392204

[35] F. Paruk , J. M. Chausse , J. Emerg. Crit. Care Med. 2019, 3, 47.

[36] J. Li , Y. Li , B. Zhang , X. Shen , H. Zhao , Exp. Neurol. 2016, 284, 106. 27554829 10.1016/j.expneurol.2016.08.010

[37] H. Zhao , B. L. Zhang , S. J. Yang , B. Rusak , Behav. Brain Res. 2015, 277, 89. 25234226 10.1016/j.bbr.2014.09.016

[38] L. Durieux , V. Mathis , K. Herbeaux , M. A. Muller , A. Barbelivien , C. Mathis , R. Schlichter , S. Hugel , M. Majchrzak , L. Lecourtier , Brain Struct. Funct. 2020, 225, 2029. 32642914 10.1007/s00429-020-02107-5

[39] M. Herkenham , W. J. Nauta , J. Comp. Neurol. 1979, 187, 19. 226566 10.1002/cne.901870103

[40] Y. Matsuda , K. Fujimura , Brain Res. Bull. 1992, 28, 743. 1617458 10.1016/0361-9230(92)90254-u

[41] T. Yamaguchi , H. L. Wang , X. Li , T. H. Ng , M. Morales , J. Neurosci. 2011, 31, 8476. 21653852 10.1523/JNEUROSCI.1598-11.2011 PMC6623324

[42] A. Tchenio , S. Lecca , K. Valentinova , M. Mameli , Nat. Commun. 2017, 8, 1135. 29074844 10.1038/s41467-017-01192-1 PMC5658350

[43] M. Maletic‐Savatic , R. Malinow , K. Svoboda , Science 1999, 283, 1923. 10082466 10.1126/science.283.5409.1923

[44] H. Kasai , M. Fukuda , S. Watanabe , A. Hayashi‐Takagi , J. Noguchi , Trends Neurosci. 2010, 33, 121. 20138375 10.1016/j.tins.2010.01.001

[45] H. Kasai , N. E. Ziv , H. Okazaki , S. Yagishita , T. Toyoizumi , Nat. Rev. Neurosci. 2021, 22, 407. 34050339 10.1038/s41583-021-00467-3

[46] I. Bethus , D. Tse , R. G. Morris , J. Neurosci. 2010, 30, 1610. 20130171 10.1523/JNEUROSCI.2721-09.2010 PMC6633999

[47] D. De Bundel , T. Femenía , C. M. DuPont , Å. Konradsson‐Geuken , K. Feltmann , B. Schilström , M. Lindskog , Int. J. Neuropsychopharmacol. 2013, 16, 2041. 23672849 10.1017/S1461145713000370

[48] R. R. Huang , W. Hu , Y. Y. Yin , Y. C. Wang , W. P. Li , W. Z. Li , Int. J. Mol. Med. 2015, 35, 553. 25482165 10.3892/ijmm.2014.2026

[49] H. Tan , J. Cao , J. Zhang , Z. Zuo , J. Neuroinflammation 2014, 11, 93. 24884762 10.1186/1742-2094-11-93 PMC4046437

[50] B. Zheng , R. Lai , J. Li , Z. Zuo , Brain, Behav., Immun. 2017, 61, 365. 28089560 10.1016/j.bbi.2017.01.005 PMC5316360

[51] A. Reiner , J. Levitz , Neuron 2018, 98, 1080. 29953871 10.1016/j.neuron.2018.05.018 PMC6484838

[52] Biology of the NMDA Receptor, (Ed: A. M. Van Dongen ), CRC Press/Taylor & Francis, Boca Raton, FL 2009. 21204416

[53] A. Ludhiadch , R. Sharma , A. Muriki , A. Munshi , CNS Neurol. Disord.: Drug Targets 2022, 21, 52. 33583386 10.2174/1871527320666210212141232

[54] T. R. Guilarte , J. L. McGlothan , Mol. Brain Res. 2003, 113, 37. 12750004 10.1016/s0169-328x(03)00083-4

[55] Y. Dong , A. V. Kalueff , C. Song , J. Neuroimmunol. 2017, 307, 7. 28495142 10.1016/j.jneuroim.2017.03.005

[56] S. A. Oakes , F. R. Papa , Annu. Rev. Pathol. 2015, 10, 173. 25387057 10.1146/annurev-pathol-012513-104649 PMC5568783

[57] M. J. Berridge , P. Lipp , M. D. Bootman , Nat. Rev. Mol. Cell Biol. 2000, 1, 11. 11413485 10.1038/35036035

[58] D. Lindholm , L. Korhonen , O. Eriksson , S. Kõks , Front. Cell Dev. Biol. 2017, 5, 48. 28540288 10.3389/fcell.2017.00048 PMC5423914

[59] P. Walter , D. Ron , Science 2011, 334, 1081. 22116877 10.1126/science.1209038

[60] S. E. Logue , P. Cleary , S. Saveljeva , A. Samali , Apoptosis 2013, 18, 537. 23430059 10.1007/s10495-013-0818-6

[61] Y. Ma , J. W. Brewer , J. A. Diehl , L. M. Hendershot , J. Mol. Biol. 2002, 318, 1351. 12083523 10.1016/s0022-2836(02)00234-6

[62] J. Min , Z. Lai , H. Wang , Z. Zuo , CNS Neurosci. Ther. 2022, 28, 619. 34882968 10.1111/cns.13777 PMC8928916

[63] K. Maekawa , T. Baba , S. Otomo , S. Morishita , N. Tamura , PLoS One 2014, 9, 87375. 10.1371/journal.pone.0087375 PMC3903674 24475280

[64] M. Antunes , G. Biala , Cognit. Process. 2012, 13, 93. 22160349 10.1007/s10339-011-0430-z PMC3332351

[65] P. A. Lipton , H. Eichenbaum , Neural. Plast. 2008, 2008, 258467. 18615199 10.1155/2008/258467 PMC2443546

[66] C. S. Rosenfeld , S. A. Ferguson , J. Visualized Exp. 2014, 84, e51194. 10.3791/51194 PMC4140524 24637673

[67] N. Chaaya , A. R. Battle , L. R. Johnson , Neurosci. Biobehav. Rev. 2018, 92, 43. 29752958 10.1016/j.neubiorev.2018.05.013

[68] Y. M. Yoon , J. H. Lee , S. P. Yun , Y. S. Han , C. W. Yun , H. J. Lee , H. Noh , S. J. Lee , H. J. Han , S. H. Lee , Sci. Rep. 2016, 6, 39838. 28004805 10.1038/srep39838 PMC5177936

[69] X. Song , M. O. Jensen , V. Jogini , R. A. Stein , C. H. Lee , H. S. McHaourab , D. E. Shaw , E. Gouaux , Nature 2018, 556, 515. 29670280 10.1038/s41586-018-0039-9 PMC5962351

[70] W. Shan , J. Li , W. Xu , H. Li , Z. Zuo , Cell. Mol. Life Sci. 2019, 76, 1381. 30666338 10.1007/s00018-019-03007-6 PMC6421091

[71] H. Li , J. Yin , L. Li , J. Deng , C. Feng , Z. Zuo , Neurobiol. Dis. 2013, 54, 216. 23313315 10.1016/j.nbd.2012.12.014 PMC3628970

[72] J. Zhang , H. Tan , W. Jiang , Z. Zuo , Anesthesiology 2014, 121, 773. 25251457 10.1097/ALN.0000000000000352 PMC4176814

