# Supplementary material for: Activation of the Lateral Habenula‐Ventral Tegmental Area Neural Circuit Contributes to Postoperative Cognitive Dysfunction in Mice
Source: Adv Sci (Weinh). 2022 May 26;9(22):2202228. doi: 10.1002/advs.202202228 (PMC9353455; doi:10.1002/advs.202202228)
Supplement: Supplementary file 1 — Supporting Information [file ADVS-9-2202228-s001.pdf]

Supporting information

Figure S1: Indication of success of viral transfection into the LHb. a) Representative images showing the expression of mCherry whose code was carried by the viral vector that was injected into the LHb. Scale bar: 100 (the first panel on the left) and 10 μm (the other panels). b) Co-localization of mCherry with VGAT or vGluT2 in the VTA. Viral vector was injected into the LHb. Scale bar: 10 μm. c) Transfection efficiency to the neurons in the LHb. Left panel: Representative immunofluorescent images of brain sections of mice receiving injection of AAV-hSyn-hM4D(Gi)-mCherry or AAV-hSyn-mCherry into the LHb. Scale bar: 100 μm. Right panel: Quantitative data. Data are presented as mean ± S.D. with the presentation of data of each individual animal (n = 4).

Figure S2. Examination of neuronal projections to the VTA by using retrograde viruses carrying *gfp* code in mice with surgery. Left: schematic presentation of the viral injection. Right: representative images of GFP and c-Fos expression in a brain section in low magnification (top panel) and in LHb, hippocampus and prefrontal cortex (bottom three panels, scale bars: 100 μm). The area surrounded by white dashed lines in the top panel is where VTA locates.

Figure S3. Areas stained by Evans blue injected into the LHb (a) or VTA (b). Left panel: an enlarged image with labeling of brain regions. Scale bar: 500 μm. Right panels: images of consecutive sections of every 100 μm.

Figure S4. Inhibition or chemical damage of LHb neurons reduced the number of c-Fos positive cells in the RMTg after surgery. a) Representative c-Fos immunofluorescence images of the LHb, VTA and RMTg. Scale bar: 100 μm. b) Number of c-Fos-positive neurons in the RMTg after LHb neurons were inhibited. c-e) Number of c-Fos-positive neurons in the LHb, VTA and RMTg after LHb neurons were inhibited retrogradely via the projections from LHb to VTA. f) Representative c-Fos immunofluorescence images of the RMTg. Scale bar: 100 μm. g) Number of c-Fos-positive cells in the RMTg. Data are presented as mean ± S.D. with the presentation of data of each individual animal (n = 6 for panels b to e, 5 for panel g). Results were analyzed by one-way ANOVA followed with Tukey test. * $P < 0.05$ for the comparison, ** $P < 0.01$ for the comparison. IBO: ibotenic acid.

Figure S5. Diagram of time-line of the six sets of experiments. BM: Barnes maze test, BMlt: Barnes maze long-term memory test, BMst: Barnes maze short-term memory test, C21: compound 21, FC: fear conditioning test, IBO: ibotenic acid, LHb: lateral habenula, NOR: novel

object recognition test, NS; normal saline, OF: open field test, TUDCA: tauroursodeoxycholic acid.

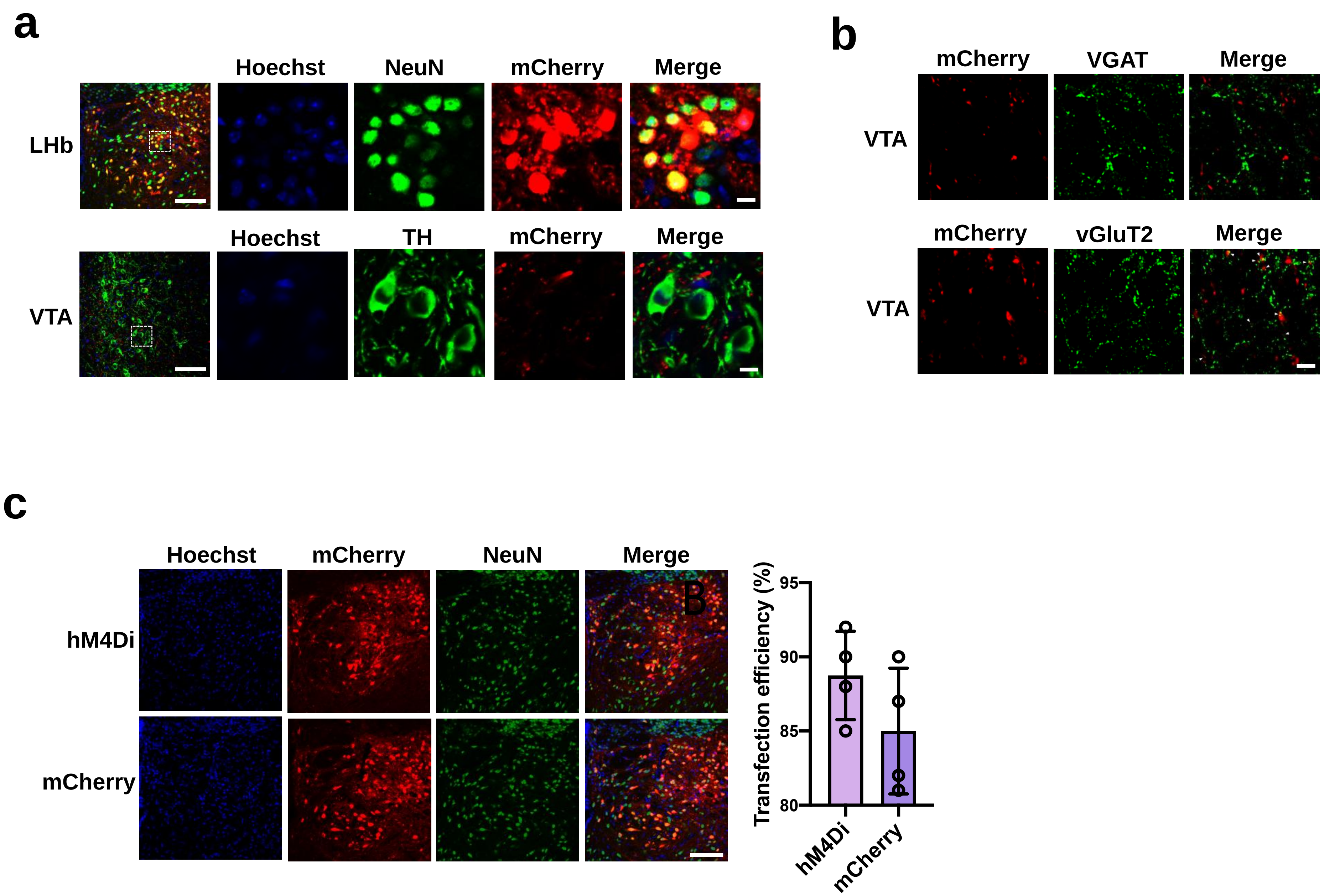
a
Hoechst
NeuN
mCherry
Merge
LHb
Hoechst
TH
mCherry
Merge
VTA
b
mCherry
VGAT
Merge
VTA
mCherry
vGluT2
Merge
VTA
c
Hoechst
mCherry
NeuN
Merge
hM4Di
mCherry
Transfection efficiency (%)
95
90
85
80
hM4Di
mCherry

Figure S1

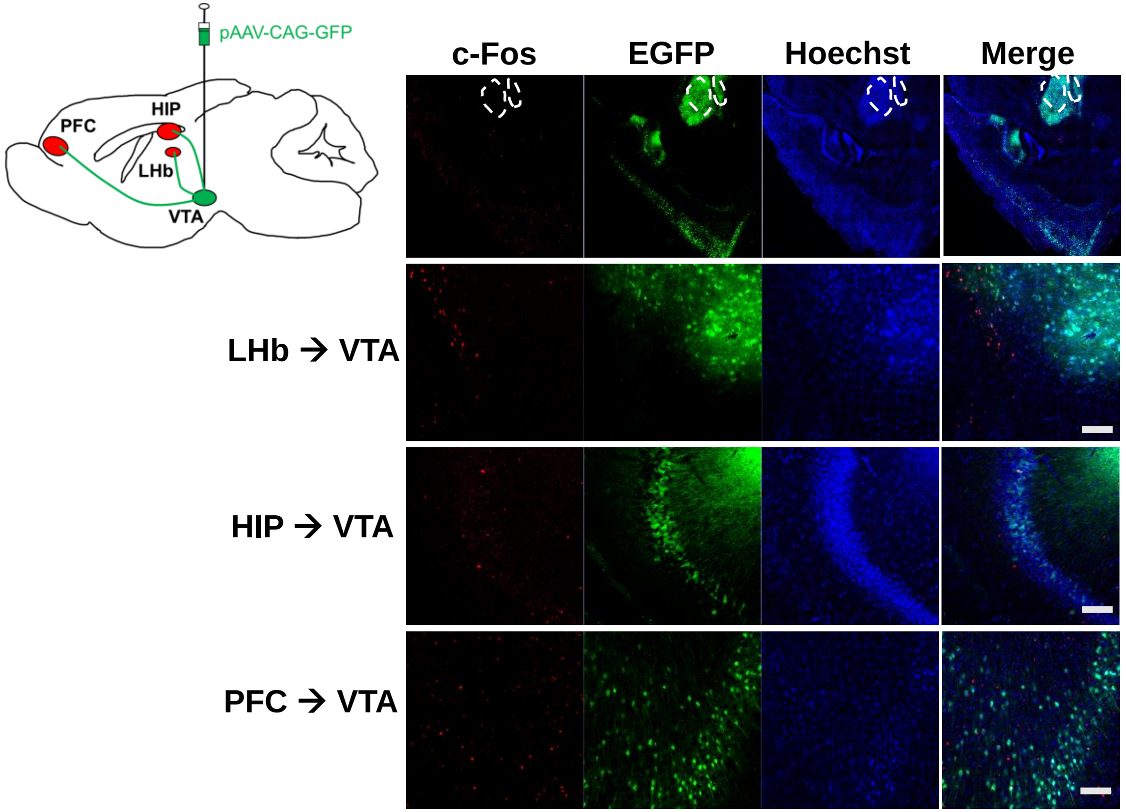
pAAV-CAG-GFP
PFC
HIP
LHb
VTA
c-Fos
EGFP
Hoechst
Merge
LHb → VTA
HIP → VTA
PFC → VTA

Figure S2

a

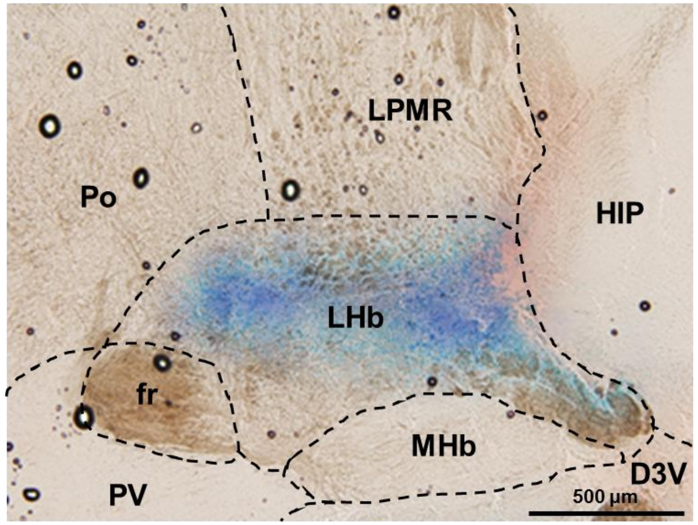

Po: posterior thalamic nuclear group; LPMR: lateral posterior thalamic nucleus, mediorostral part; HIP: hippocampus; LHb: lateral habenular nucleus; MHb: medial habenular nucleus; D3V: dorsal 3rd ventricle; fr: fasciculus retroflexus; PV: paraventricular thalamic nucleus

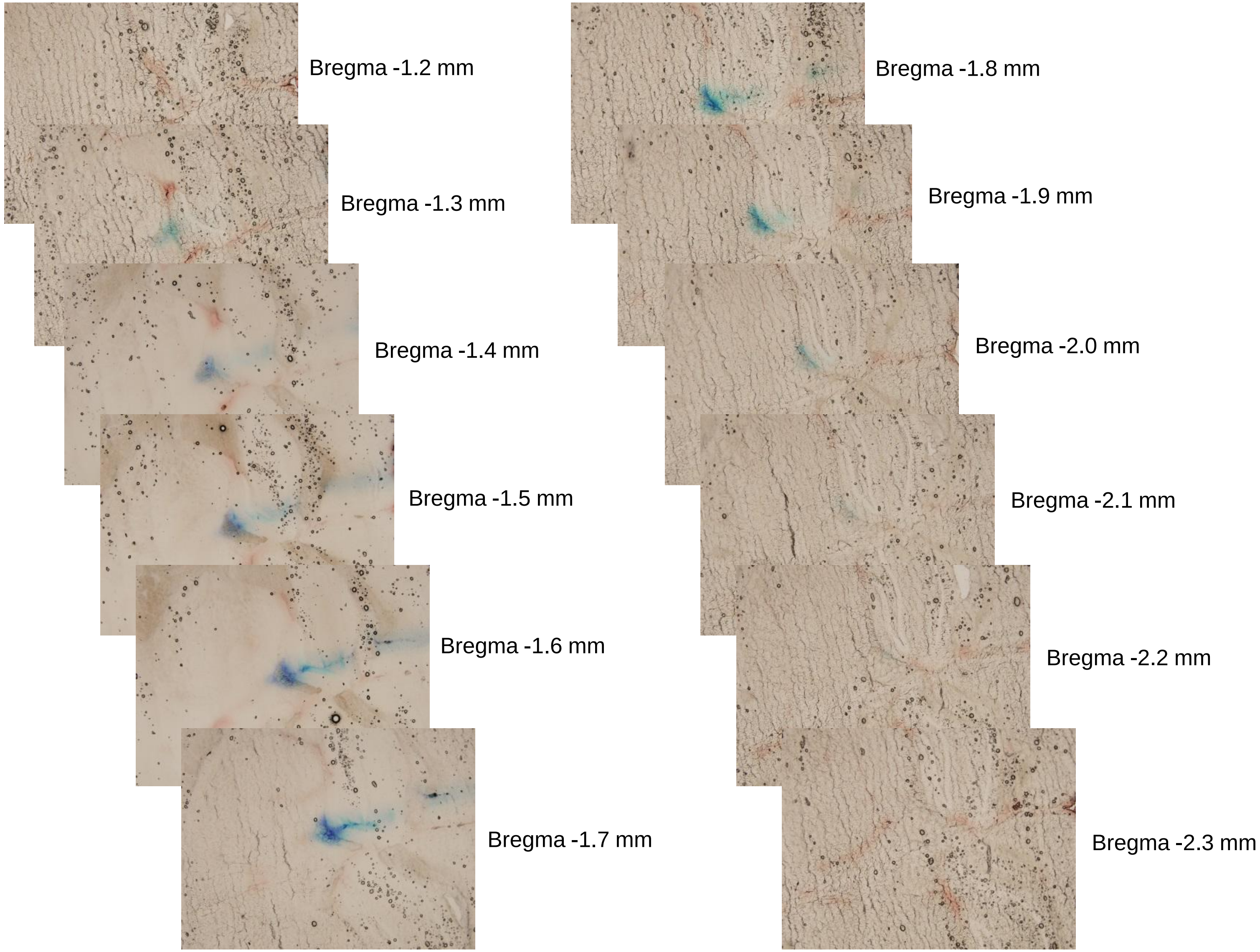

Figure S3a

b

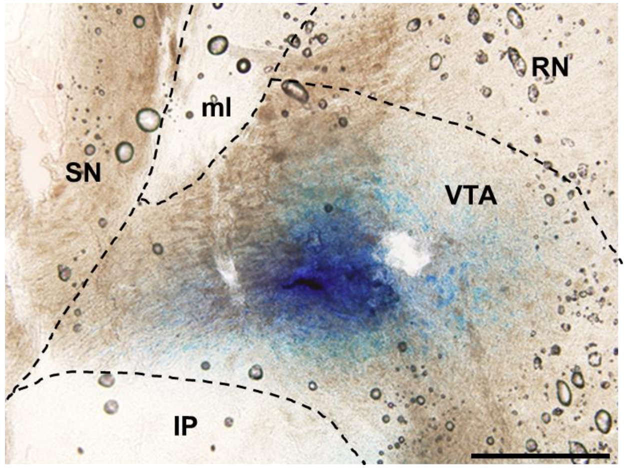

SN: substantia nigra; ml: medial lemniscus; RN: red nucleus; VTA: ventral tegmental area; IP: interpeduncular nucleus

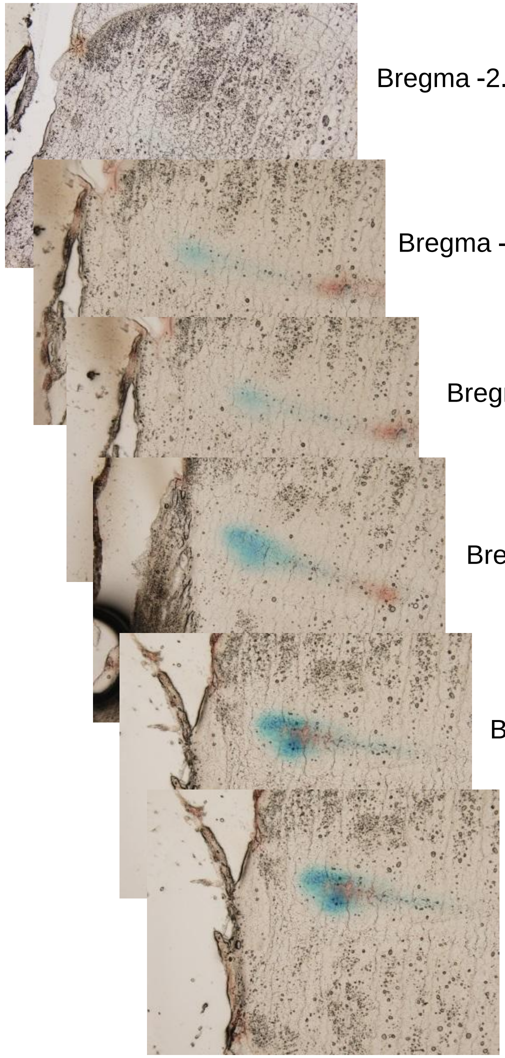

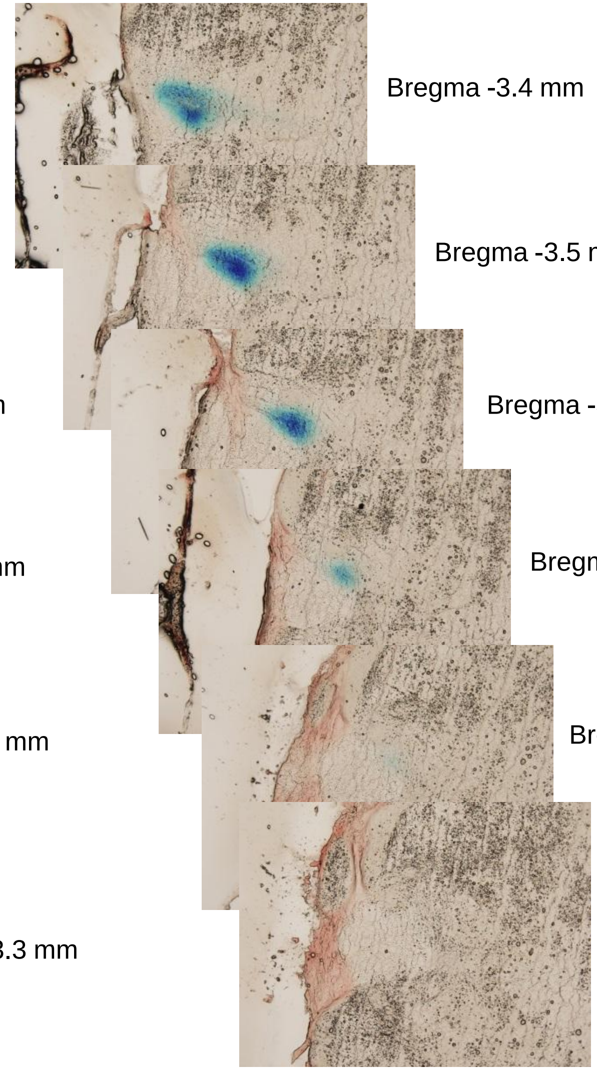

Figure S3b

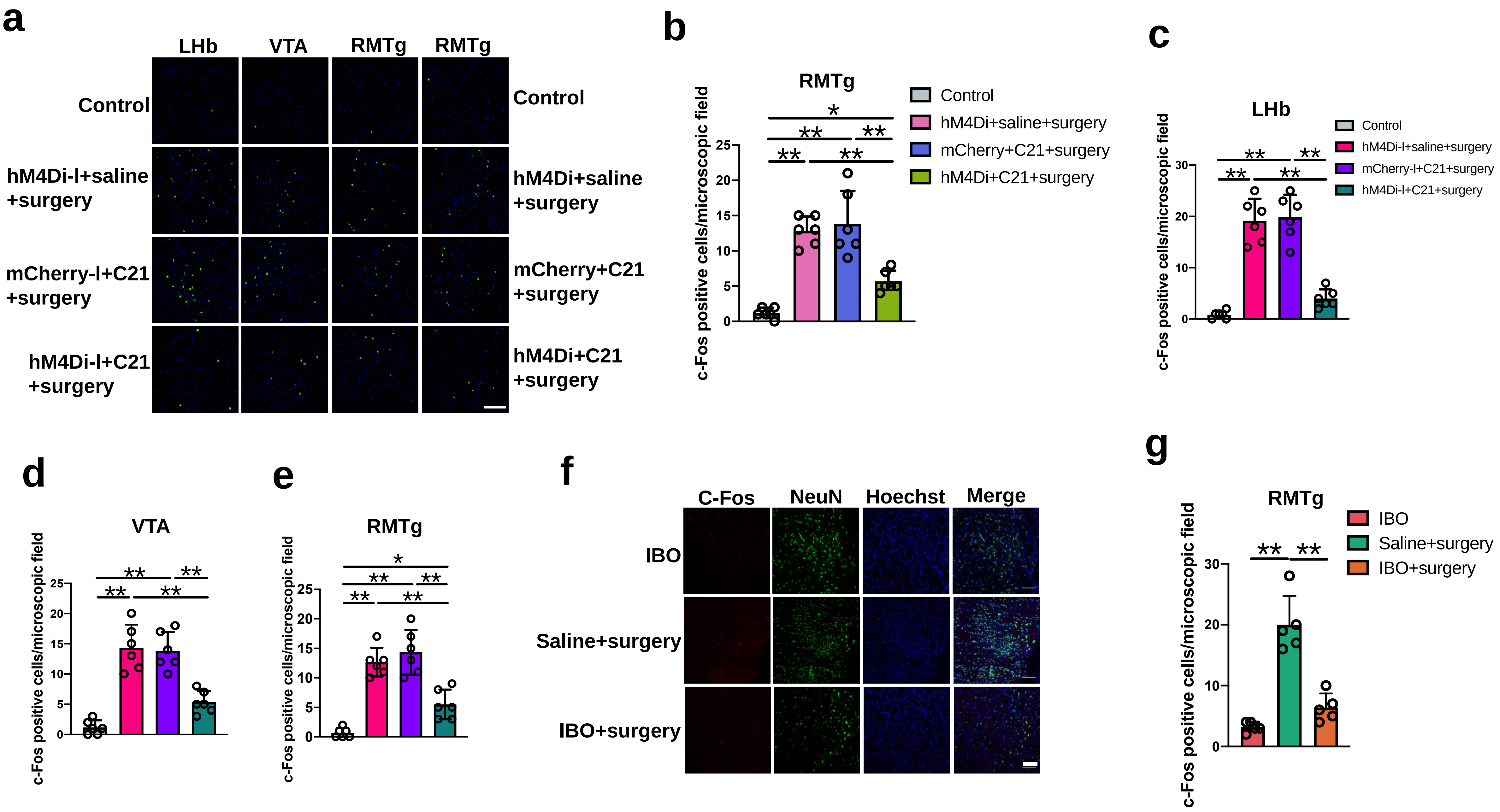
a
LHb
VTA
RMTg
RMTg
Control
hM4Di-l+saline +surgery
mCherry-l+C21 +surgery
hM4Di-l+C21 +surgery
Control
hM4Di+saline +surgery
mCherry+C21 +surgery
hM4Di+C21 +surgery
b
RMTg
c-Fos positive cells/microscopic field
Control
hM4Di+saline+surgery
mCherry+C21+surgery
hM4Di+C21+surgery
c
LHb
c-Fos positive cells/microscopic field
Control
hM4Di-l+saline+surgery
mCherry-l+C21+surgery
hM4Di-l+C21+surgery
d
VTA
c-Fos positive cells/microscopic field
e
RMTg
c-Fos positive cells/microscopic field
f
C-Fos
NeuN
Hoechst
Merge
IBO
Saline+surgery
IBO+surgery
g
RMTg
c-Fos positive cells/microscopic field
IBO
Saline+surgery
IBO+surgery

Figure S4

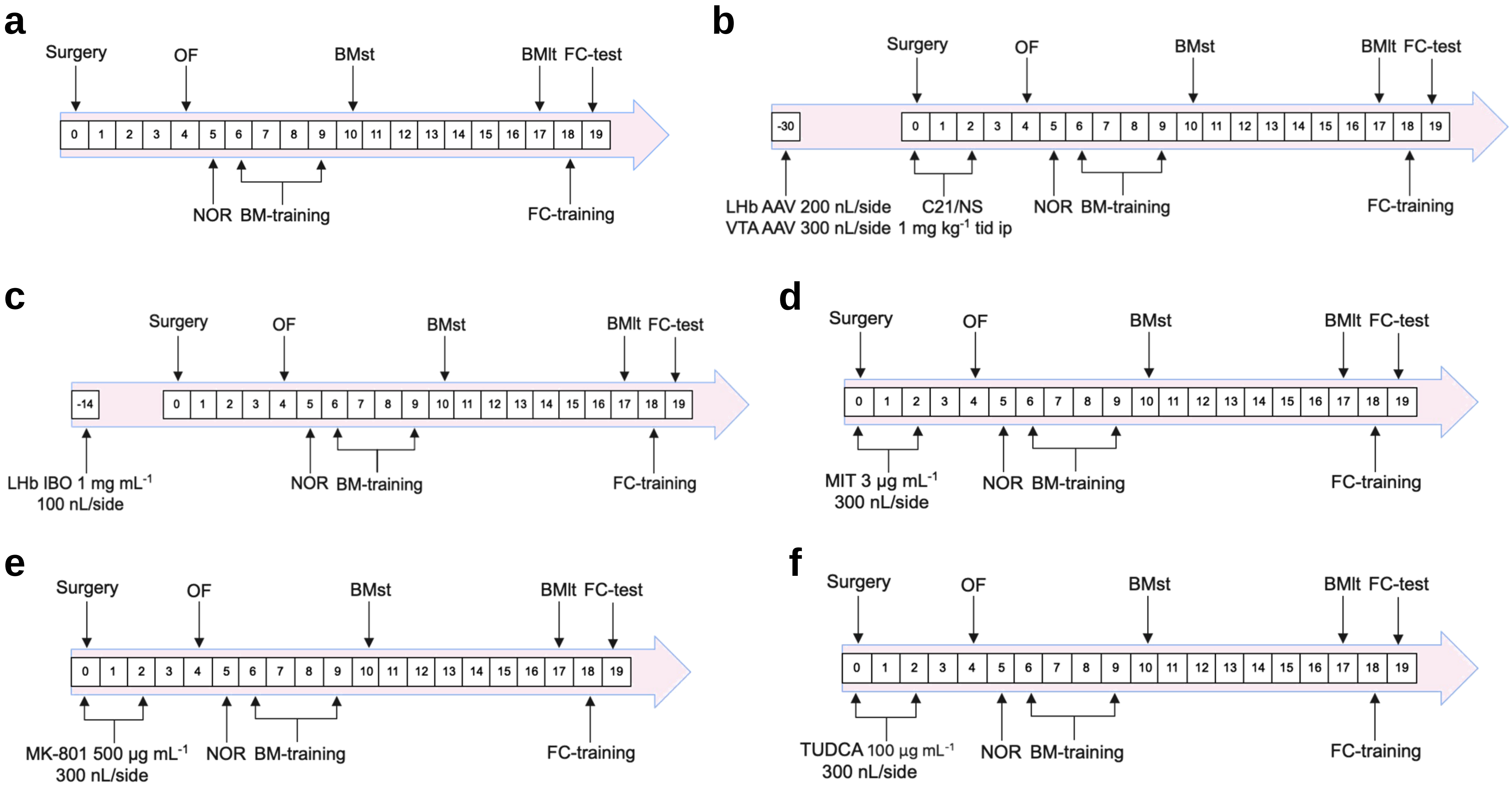
a
Surgery
OF
BMst
BMlt
FC-test
0 1 2 3 4 5 6 7 8 9 10 11 12 13 14 15 16 17 18 19
NOR
BM-training
FC-training
b
Surgery
OF
BMst
BMlt
FC-test
-30
0 1 2 3 4 5 6 7 8 9 10 11 12 13 14 15 16 17 18 19
LHb AAV 200 nL/side
VTA AAV 300 nL/side
C21/NS
1 mg kg-1 tid ip
NOR
BM-training
FC-training
c
Surgery
OF
BMst
BMlt
FC-test
-14
0 1 2 3 4 5 6 7 8 9 10 11 12 13 14 15 16 17 18 19
LHb IBO 1 mg mL-1
100 nL/side
NOR
BM-training
FC-training
d
Surgery
OF
BMst
BMlt
FC-test
0 1 2 3 4 5 6 7 8 9 10 11 12 13 14 15 16 17 18 19
MIT 3 µg mL-1
300 nL/side
NOR
BM-training
FC-training
e
Surgery
OF
BMst
BMlt
FC-test
0 1 2 3 4 5 6 7 8 9 10 11 12 13 14 15 16 17 18 19
MK-801 500 µg mL-1
300 nL/side
NOR
BM-training
FC-training
f
Surgery
OF
BMst
BMlt
FC-test
0 1 2 3 4 5 6 7 8 9 10 11 12 13 14 15 16 17 18 19
TUDCA 100 µg mL-1
300 nL/side
NOR
BM-training
FC-training

Figure S5
